# Maternal stress programs a demasculinization of glutamatergic transmission in stress-related brain regions of aged rats

**DOI:** 10.1007/s11357-021-00375-5

**Published:** 2021-05-13

**Authors:** Remy Verhaeghe, Vance Gao, Sara Morley-Fletcher, Hammou Bouwalerh, Gilles Van Camp, Francesca Cisani, Ferdinando Nicoletti, Stefania Maccari

**Affiliations:** 1 IRCCS Neuromed, (IS), Pozzilli, Italy; 2 Unité de Glycobiologie Structurale et Fonctionnelle, University of Lille, CNRS, UMR 8576, UGSF, F-59000 Lille, France; 3 International Associated Laboratory (LIA) “Perinatal Stress and Neurodegenerative Diseases”, University of Lille – CNRS, UMR 8576, Lille, France; 4 International Associated Laboratory (LIA) “Perinatal Stress and Neurodegenerative Diseases”, Sapienza University of Rome – IRCCS Neuromed, Rome, Italy; 5 Department of Pharmacy (DiFar), University of Genoa, Viale Cembrano 4, 16148 Genoa, Italy; 6 Department of Physiology and Pharmacology “V. Erspamer”, Sapienza University of Rome, Rome, Italy; 7 Department of Science and Medical-Surgical Biotechnology, Sapienza University of Rome, Rome, Italy

**Keywords:** Early life stress, Sex differences, Brain aging, Behaviors, Hormones

## Abstract

Brain aging may be programmed by early-life stress. Aging affects males and females differently, but how perinatal stress (PRS) affects brain aging between sexes is unknown. We showed behavioral and neurobiological sex differences in non-stressed control rats that were strongly reduced or inverted in PRS rats. In particular, PRS decreased risk-taking behavior, spatial memory, exploratory behavior, and fine motor behavior in male aged rats. In contrast, female aged PRS rats displayed only increased risk-taking behavior and reduced exploratory behavior. PRS induced large reductions in the expression of glutamate receptors in the ventral and dorsal hippocampus and prefrontal cortex only in male rats. PRS also reduced the expression of synaptic vesicle-associated proteins, glucocorticoid receptors (GR), and mineralocorticoid receptors (MR) in the ventral hippocampus of aged male rats. In contrast, in female aged rats, PRS enhanced the expression of MRs and brain-derived neurotrophic factor (BDNF) in the ventral hippocampus and the expression of glial fibrillary acidic protein (GFAP) and BDNF in the prefrontal cortex. A common PRS effect in both sexes was a reduction in exploratory behavior and metabotropic glutamate (mGlu2/3) receptors in the ventral hippocampus and prefrontal cortex. A multidimensional analysis revealed that PRS induced a demasculinization profile in glutamate-related proteins in the ventral and dorsal hippocampus and prefrontal cortex, as well as a demasculinization profile of stress markers only in the dorsal hippocampus. In contrast, defeminization was observed only in the ventral hippocampus. Measurements of testosterone and 17-β-estradiol in the plasma and aromatase in the dorsal hippocampus were consistent with a demasculinizing action of PRS. These findings confirm that the brains of males and females differentially respond to PRS and aging suggesting that females might be more protected against early stress and age-related inflammation and neurodegeneration. Taken together, these results may contribute to understanding how early environmental factors shape vulnerability to brain aging in both sexes and may lay the groundwork for future studies aimed at identifying new treatment strategies to improve the quality of life of older individuals, which is of particular interest given that there is a high growth of aging in populations around the world.

**Supplementary Information:**

The online version contains supplementary material available at 10.1007/s11357-021-00375-5.

## Introduction

Aging is characterized by a progressive loss of physiological integrity, leading to impaired function and increased risk of death ([1]). Aging affects males and females differently [2–4]. This is extremely important if we consider, for example, the current global coronavirus disease 2019 (COVID-19) pandemic, where aging is known to be a key risk factor for severe COVID-19 [5]. Males have been found to be more vulnerable to COVID-19 than females [6]. Furthermore, stress is well-known to contribute to the variability of the aging process and the development of age-related central nervous system (CNS) disorders [7–9]. The stress hormones, glucocorticoids, regulate a cohort of physiological functions, such as intermediary metabolism and the immune system, and influence development, growth, and aging [10]. Excess of glucocorticoids, as occurs during chronic stress, may alter physiological aging [11–13]. Studies in animals and humans have shown that stressful events during critical periods of brain development cause lifelong alterations in brain programming [14, 15]. Early-life stress impacts cognitive processing during aging, as demonstrated in middle-aged and aged male rodents performing the Y-maze test and touch panel operant task [16, 17]. Perinatal stress (PRS) affects other aging-related processes in adult male rats, as indicated by an increased expression of pro-inflammatory markers [18], inhibition of neurogenesis in the hippocampus [19], and accelerated aging of the hypothalamic–pituitary–adrenal (HPA) axis [16, 20]. Glutamate, the most abundant excitatory neurotransmitter in the CNS, is deeply involved in stress-related disorders [21, 22]. Glutamatergic pyramidal neurons that mediate cortico-cortical connections between the association cortices and excitatory hippocampal connections are particularly vulnerable to aging [23, 24]. Importantly, glutamate plays a key role in the programming effects induced by PRS. Indeed, PRS greatly reduces glutamate release in the ventral hippocampus of adult male rats, an effect associated with reduced expression of synaptic vesicle-associated proteins [25–27]. Enhancing glutamate release through a cocktail of metabotropic glutamate receptor (mGlu)2/3 and GABA_B_ receptor antagonists reverses the alterations in risk-taking behavior in PRS rats [26], reinforcing the idea that impairment of the glutamatergic synapse in the ventral hippocampus lies at the core of the pathological phenotype triggered by PRS.

The decline in gonadal hormones that occurs with aging is associated with stress deregulation in males and females [8, 28]. Furthermore, gonadal hormones are altered by PRS, as demonstrated by increased plasma dihydrotestosterone levels in adult male PRS rats and lower plasma estradiol (E_2_) levels in adult PRS females [29]. Interestingly, a decrease in estradiol levels was still observed in middle-aged female rats, suggesting that PRS accelerates the aging-related-disruption of the estrous cycle [30, 31]. These hormonal changes are associated with sex differences in the behavior of adult PRS rats. For example, PRS decreases risk-taking behavior (measured in the elevated plus maze) in males but not in females [32] and differentially affects addictive behavior in males and females [29]. Other studies using prenatal stress paradigms have shown disruptions of sex differences in behavior, morphology, sex hormones, and gene expression profiles [33–37]. This suggests that the early perinatal period represents a specific window of sensitivity during which offspring are susceptible to the programming effects of PRS combined with sex differences. Interestingly, specific patterns of demasculinization have already been reported in fetal and adult life at physiological and behavioral levels [37–43]. However, very little is known about the brain-gonadal axis. For example, a possible link between plasma testosterone levels and glial fibrillary acidic protein (GFAP) expression in the CNS has been shown. In particular, previous studies have shown an inverse correlation between plasma testosterone levels and the age-dependent increase in GFAP messenger ribonucleic acid (mRNA) levels in the rat and human brain [44]. In addition, testosterone replacement lowered GFAP levels in the cerebellum of castrated male aged rats [45]. However, most studies concerning stressful perinatal events have been carried out in adult males. To our knowledge, there are no studies on the effects of PRS in elderly male and female rats and the effect on the brain-gonadal axis. Thus, we investigated the long-lasting programming effects of PRS in both sexes on behaviors and biochemical markers of glutamatergic transmission, stress, neuroplasticity, and sex hormones. To this aim, we used the PRS rat model, in which exposure of pregnant mothers to restraint stress reduces maternal behavior, to study neuroplasticity in brain regions sensitive to stress (ventral and dorsal hippocampus, prefrontal cortex and striatum), and cognitive and motor behaviors in male and female rats aged 21–22 months.

## Materials and methods

### Experimental design

**Fig. 1:**
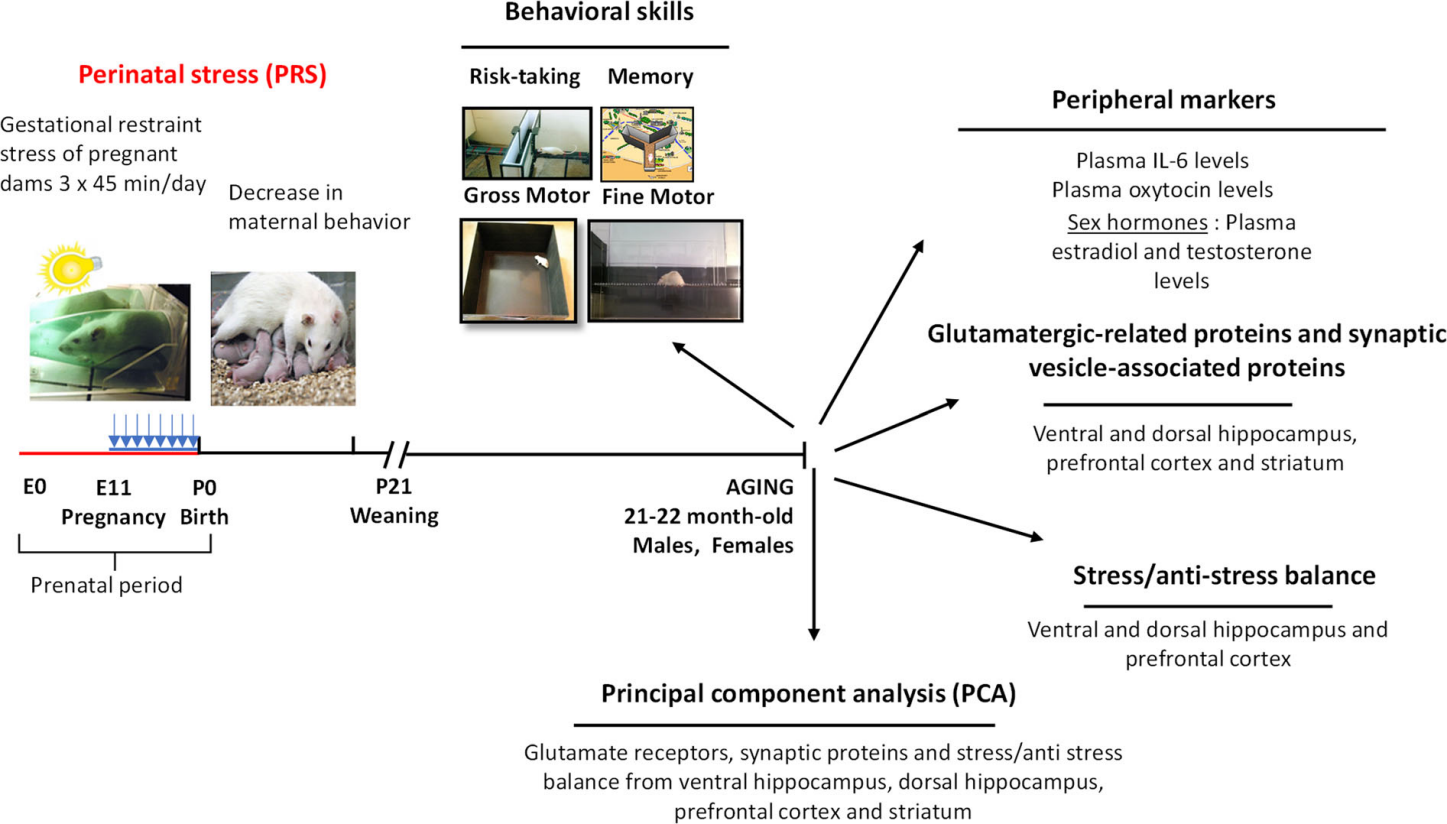
Experimental design and timeline. Induction of PRS and maternal behavior analysis in the first *postpartum* week were followed by behavioral and biochemical measurements in the 21–22 month old male and female progeny as indicated. Several multidimensional analyses have been performed

The experimental timeline is shown in Fig. 1. After the PRS procedure, which consisted of restraint stress of pregnant mothers and the reduction of maternal behavior in the first postpartum week, behavioral and biochemical measurements in the hippocampus (ventral and dorsal), prefrontal cortex, and striatum in both male and female rats (21–22 months) were studied. Behavioral tests were performed when the rats were 21 months old. One week after the last behavioral test, the brain structures and blood (plasma and serum) were collected. All experiments followed the rules of the European Communities Council Directive 86/609/EEC. The Local Committee CEEA-75 (Comité d’Ethique en Experimentation Animale Nord-Pas-de-Calais, 75) approved the experimental procedures.

### Animals

Thirty nulliparous female Sprague Dawley rats, weighing approximately 250 g, were purchased from Charles River (France) and housed under standard conditions with a 12-h light/dark cycle. After group housing (five females/cage) for 2 weeks, each female was individually housed for 1 week with a sexually experienced male rat. Following this, a gain of at least 10 g was considered to indicate pregnancy. For the offspring, 8–12 animals per group were used for each behavioral test and four to nine animals per group for biochemical analysis (except for vesicular glutamate transporter 1 (vGLUT1) and GluA3 immunoblots in the ventral hippocampus, *n* = 3–4 animals per group and *n* = 2–4 animals per group, respectively).

### Stress procedure

The stress procedure was performed on one breeding set, using 30 females (15 CONTs and 15 stressed dams). PRS rats, that is, the adult offspring of dams exposed to multiple episodes of restraint stress during pregnancy causing reduced maternal care were obtained according to our standard protocol [20], as shown in Fig. 1. Briefly, from day 11 of pregnancy until delivery, pregnant females were subjected to restraint in a transparent plastic cylinder and exposed to bright light during three daily sessions of 45 min. Control pregnant females were left undisturbed in their home cages and were handled once per week. After weaning, male and female offspring from the litter with a balanced sex ratio were used for the experiments. Animals were housed in groups of two or three and maintained under similar environmental conditions during their entire life span; 21–22-month old rats were used in all experiments. The animals were weighed at 21 months prior to the behavioral assessment.

### Maternal behavior

Maternal behavior was monitored for 24 h every day during the first 7 postpartum days. Constant monitoring was performed with small infrared cameras placed on the animal cage rack where cages containing lactating females were placed. Within each observation period, the behavior of each mother was scored every minute from postpartum day 1 to day 7 (60 observations/h with 2 h of observation per day, 1 h before lights off, and 1 h after lights on). The active behavior of the mother (nursing behavior, grooming, licking, and carrying pups) was scored, and the data obtained were expressed as percentages with respect to the total number of observations. Because gestational stress induces a reduction of maternal behavior [46], we refer to the whole procedure as perinatal stress (PRS).

### Behavioral studies

#### Risk-taking behavior in an elevated plus maze test and exploratory behavior

Risk-taking behavior of PRS or control progeny [27] was assessed in the elevated plus maze test (EPM) [47]. Briefly, the test was performed for 5 min early in the afternoon (between 1 and 4 pm) and began with the placement of the rat in the center of the maze with the head facing a closed arm. We used a custom-made EPM apparatus described by Vallée et al. [16], with closed and open arms of 20 × 20 cm. The closed arms’ luminosity was approximately 25 lx, and the luminosity of the open arms was approximately 50 lx. Behavior was recorded by a video camera and manually scored by a trained observer blind to the animals’ condition (PRS and control) using a software package (Noldus, The Observer®). The time spent in the open and closed arms was measured, and the percentage of time spent in the open arms was calculated and analyzed as risk-taking behavior. The number of closed arms entries was analyzed as exploratory behavior.

#### Fine motor skills in the ladder rung-walking test

The horizontal ladder rung-walking test apparatus [48] consisted of side walls made of clear plexiglass and metal rungs (3 mm diameter), which could be inserted to create a floor with a minimum distance of 1 cm between rungs. The sidewalls were 1 m long and 19 cm high, as measured from the height of the rungs. The ladder was elevated 30 cm above the ground with a refuge (home cage) at the end. Varying the position of the metal rungs modified the difficulty of the task. A regular pattern of the rungs allowed the animals to learn the pattern over several training sessions and to anticipate the position of the rungs. To increase the test’s difficulty, after the training sessions, an irregular pattern was created to analyze how rats managed to cross the ladder. The test was recorded using two video cameras. One video camera was placed in front of the first half of the ladder at a slight ventral angle, and the other was placed in front of the second half of the ladder at a slight ventral angle to precisely analyze the misplacement of the rat paw. Following the video recording, the foot faults were scored. A score of one was assigned when the rat’s paw was misplaced, and a score of two was assigned when the rat’s paw severely slipped or missed. Then, the percentage increase in errors between the last training and the test with the irregular pattern was calculated as follows: error score irregular pattern − error score last training/error score last training * 100.

#### Spatial recognition memory in Y-maze and exploratory behavior

Spatial recognition memory was measured in a two-trial memory task in a Y-maze [16] made of gray plastic with three identical arms (50 cm) enclosed with 32-cm-high side walls and illuminated by dim light (40 lx). Each arm was equipped with two infrared beams, one at each end of the arm. The maze floor was covered with rat odor-saturated sawdust, and the sawdust was mixed between each session to eliminate olfactory cues. Visual cues were placed in the testing room and kept constant during the behavioral testing sessions. The task consisted of two trials separated by a time interval. In the first trial (acquisition phase), one arm of the Y-maze was closed, and animals could visit the two other arms for 5 min. During the intertrial interval (ITI), rats were housed in their home cages, which were different from the test room. During the second trial (retention phase), animals had free access to the three arms and were again allowed to explore the maze for 5 min. The time spent in the novel arm (previously closed in the first trial) was calculated as a percentage of the total time spent in all three arms during the first 3 min of the second trial. This time corresponds to the maximal exploratory activity in the novel arm, which subsequently declines [49]. Time spent in the novel arm above chance (i.e., 33%) indicates spatial recognition. Memory performance was tested with an ITI of 6 h. Total entries in the different arms were analyzed for 5 min as a measure of the exploration behavior.

#### Gross motor skills-locomotor activity in the open-field arena

Exploratory behavior was evaluated by placing a rat into a corner of an open-field arena (100 × 100 × 50 cm), allowing the rat to explore the field for 10 min freely. Lightning was approximately 60 lx inside the arena. Activity and trajectory length in the open-field was recorded and quantified by Video Track® (Viewpoint, Lyon, France).

### Western blot analysis

The hippocampus (ventral and dorsal), striatum, and prefrontal cortex of control (CONT) and PRS male and female rats were rapidly dissected and immediately stored at −80 °C. Glutamate-related proteins and synaptic vesicle-associated proteins were assessed in synaptosomes. To isolate synaptosomes, tissue was manually homogenized with a potter in ten volumes of HEPES-buffered sucrose (0.32 M sucrose, 4 mM HEPES pH 7.4). All procedures were performed at 4 °C. Homogenates were centrifuged at 1000 × g for 10 min, and the resulting supernatants were centrifuged at 10,000 × g for 15 min. The pellet obtained from the second centrifugation was resuspended in ten volumes of HEPES-buffered sucrose [26]. This pellet contained the crude synaptosomal fraction. BCA assay was used to determine protein concentration. Synaptosome lysates were resuspended in Laemmli reducing buffer, and 20μg for synaptosomal fraction or 35μg from the total homogenates of each sample were loaded. The samples were loaded in two different gels. One of the samples was used as an internal control and was loaded in each gel to ensure sample homogeneity between different gels and compare samples from different gels.

Proteins were first separated by electrophoresis on sodium dodecyl sulfate-polyacrylamide gels according to their molecular weight and then transferred to nitrocellulose membranes (Bio-Rad). The transfer was performed at 4 °C in a buffer containing 35 mM Tris, 192 mM glycine, and 20% methanol. After transfer, blots were incubated in a blocking solution containing Tris-buffered saline and 5% (w/v) non-fat milk. All the following antibodies were first tested with control samples to determine the optimal conditions for use. To analyze several proteins per membrane, membranes were cut according to the molecular weight of the protein of interest. We used the following primary antibodies on synaptosomal fraction: mouse polyclonal anti-synaptosomal-associated protein, 25 kDa (SNAP25), rabbit polyclonal anti-synapsin Ia/b (1:4000; catalog #sc-20780), rabbit polyclonal anti-synaptophysin (1:8000; catalog #sc-9116), rabbit polyclonal anti-syntaxin (1:4000, catalog #sc-13994), and rabbit polyclonal anti-synapsin IIa (1:4000; catalog #sc-25538), all purchased from Santa Cruz Biotechnology; mouse monoclonal anti-rab3a (1:2000; catalog #107111), mouse monoclonal anti-Munc-18 (1:2000; catalog #116011), and mouse polyclonal anti-vesicle-associated membrane proteins (VAMP) (1:1500; catalog #104 111) which were purchased from Synaptic Systems; rabbit polyclonal anti-mGlu5 receptors (1:1000; catalog #AB5675) and rabbit polyclonal anti-mGlu2/3 receptors (1:1000; catalog #06-676), all purchased from Millipore; rabbit polyclonal anti-GluN1 (1:2000; catalog #ab109182); rabbit monoclonal anti-GluA2 (1/2000; catalog #ab206293), rabbit polyclonal anti-GluN2A (1:500; catalog #ab14596), mouse monoclonal anti-GluN2B (1/1000; catalog #ab28373), rabbit monoclonal anti-VGLUT1 (1/1000; catalog #ab180188), and rabbit polyclonal anti-vesicular glutamate transporter 2 (VGLUT2) (1/1000; catalog #ab84103) purchased from Abcam; mouse monoclonal anti-GluA3 (1/800; catalog #MAB5416) purchased from Merck; and rabbit polyclonal anti-cystine/glutamate antiporter (xCT) (1/500; catalog #KE021) purchased from TransGenic Inc. We used the following primary antibodies on total homogenates: rabbit polyclonal anti-GR (1/1000; catalog #24050-1-AP), rabbit polyclonal anti-GFAP (1/1500; catalog #16825-1-AP), and rabbit polyclonal anti-BDNF (1/1000; catalog #28205-1-AP), all purchased from Proteintech; mouse monoclonal anti-aromatase (1/500; catalog #sc-7305) and rabbit polyclonal anti-oxytocin receptor (OXTR) (1/500; catalog #sc-33209) purchased from Santa Cruz; and rabbit polyclonal anti-MR (1/1000; catalog #ab64457) from Abcam. To ensure that each lane was loaded with an equivalent amount of proteins, the blots were probed with a mouse monoclonal anti-β-actin (1:5000; catalog #A5316, Sigma). All primary antibodies were incubated overnight at 4 °C. Horseradish peroxidase-conjugated secondary anti-mouse or anti-rabbit antibodies (purchased from GE-Healthcare) were used at a dilution of 1:7500 and incubated for 1 h at room temperature. Bands were visualized with an enhanced chemiluminescence system (ECL enhancer Thermo Fisher). After immunoblotting, digitized images of bands immunoreactive for target antibodies and actin were acquired (FUSION®), and the area of immunoreactivity corresponding to each band was measured using ImageJ. Each blot was normalized to actin. The ratio of target to actin was then determined, and these values were compared for statistical significance.

### Measurement of the interleukin-6 (IL-6), oxytocin, and sex hormones levels

IL-6 (pg/mL), oxytocin (pg/mL), testosterone (ng/mL), and estradiol levels (pg/mL) were determined in the plasma extracted from blood samples. Plasma was collected using ethylenediaminetetraacetic acid (EDTA) as an anticoagulant and centrifuged for 15 min at 1000 × g at 4 °C. Plasma was stored at −20 °C until assessment. All enzyme-linked immunosorbent assay (ELISA) kits were used according to the manufacturer’s protocol. All standards, blood samples, and controls were analyzed concurrently in duplicate. The optical density of the samples was determined at 450 nm using a microplate reader (BioTek Instruments, Winooski, USA).

**Table:** 

ELISA kit	Manufacture	Sensibility range
IL-6	CUSABIO (CSB-E04640r)	0.312–20 pg/ml
Oxytocin	CUSABIO (CSB-E14197r)	7.5–600 pg/ml
Testosterone	DEMEDITECH (DEV9911 rat)	0.066–25 ng/ml
Estradiol	DEMEDITECH (DEV9999 rat)	2.5–1.280 pg/ml

### Multidimensional analyses

To see how sex and stress affect protein quantities more generally, we performed a multidimensional analysis on sets of multiple proteins or behaviors. Proteins were categorized as glutamatergic synapse proteins**,** synaptic vesicle-associated proteins, or stress/anti-stress balance-related proteins, and analyses were carried out for each protein set in each brain region.

Since protein levels tended to be correlated with each other, we used a principal components analysis (PCA) to summarize the variation in a protein set and for plotting. For the PCA, individuals who were missing more than 50% of protein measurements were excluded. Missing values were replaced with the mean of the sex-by-PRS group. To quantify distances between groups, we used the median of the Manhattan distances between each individual in one group with the centroid of another group. We created a “demasculinization score,” which represents the phenomenon in which PRS causes males to become more similar to females in various aspects. The demasculinization score was the distance from control males to control females divided by the distance from PRS males to control females. A score greater than one signifies that PRS males are more similar to control females than control males. To test the significance of demasculinization, a Mann–Whitney–Wilcoxon test was used to determine if the difference between distances was significant.

### Partial correlation

To determine significant relationships between the two measures, we calculated the correlation between the two measures after controlling for sex and group (PRS/CONT) on both measures. Generally, *Y* is a behavior, and *X* is a neurophysiological measure, such as protein or hormone levels. Moreover, *β*_1_ ≠ 0 indicates a significant correlation between *X* and *Y*.

### Statistical analysis

Behavioral and biochemical data were expressed as the mean ± standard error of the mean (SEM) and analyzed using a parametric analysis of variance (ANOVA) with group (CONT vs PRS) and sex (male and female) as independent variables. When group × sex interaction was present, post hoc comparisons were performed using the Fisher test. A *p*-value of < 0.05 was considered statistically significant. A permutational MANOVA (PERMANOVA) [50] was used to test differences between groups in ensembles of proteins.

## Results

### Sex-specific effects of PRS on behavior of aged rats

**Fig. 2:**
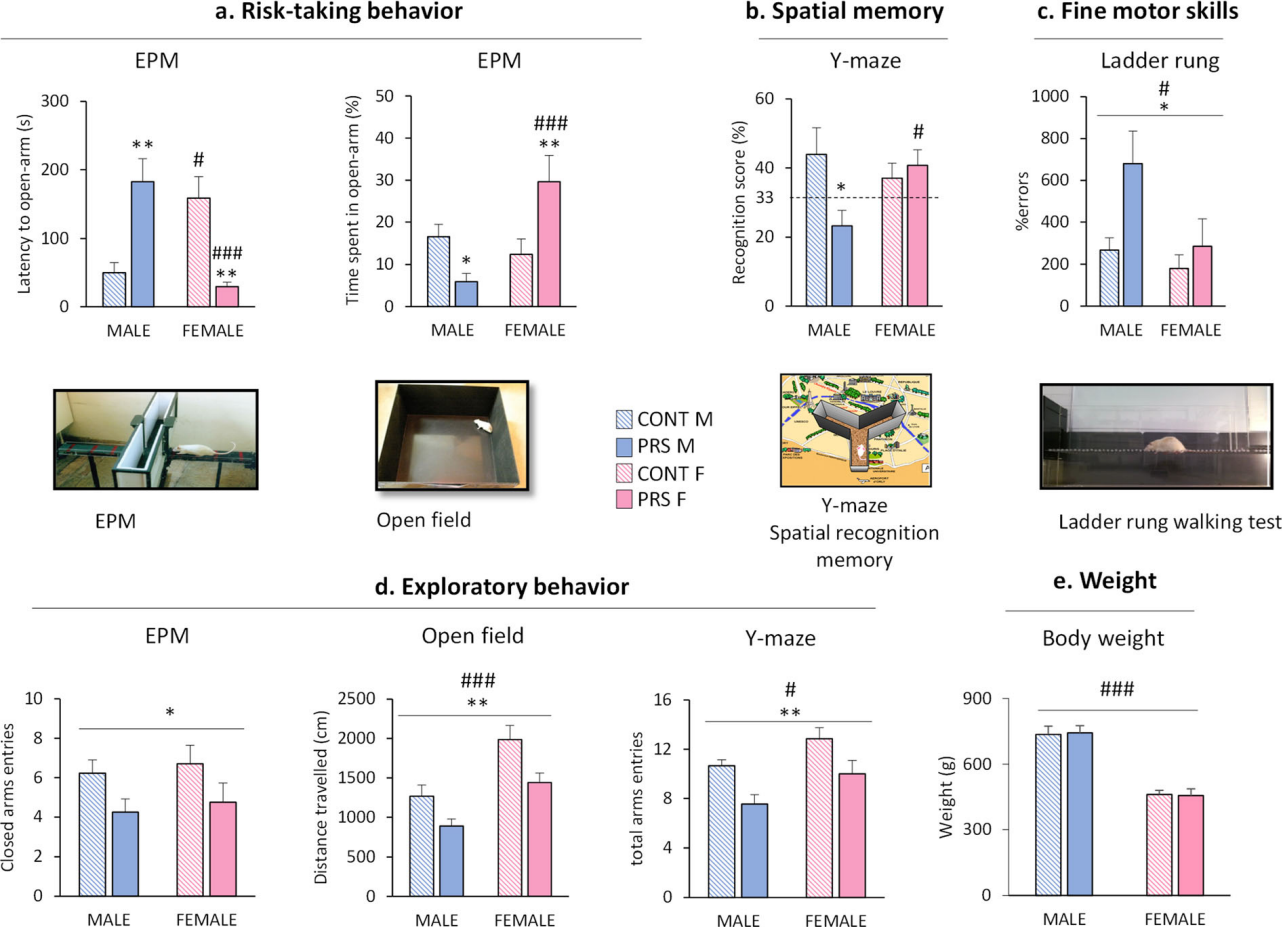
Sex-specific effects of PRS on behavior of aged rats. Risk-taking behavior in the EPM is shown in **a**. The latency to the open arm and time spent in the open arm were the two parameters analyzed. The spatial recognition memory was studied using the Y-maze. The recognition score (%) is represented in **b**. The ladder rung-walking test was used to study the fine motor skills. The percentage of errors is shown in **c**. Exploratory behavior was analyzed in the EPM test considering the closed arm entries, the open-field with the distance traveled, and the Y-maze test, where we analyzed the total arm entries (**d**). The weight of the animals before behavioral assessment (21 months old) is represented in **e**. Error bars represent the SEM. CONT vs PRS * = *p* < 0.05; ** = *p* < 0.01. Males vs females # = *p* < 0.05; ## = *p* < 0.01; ### = *p* < 0.001

For the assessment of *risk-taking behavior*, we used the EPM (Fig. 2a) to measure the latency to enter the open arm and the time spent in the open arm. We observed a sex-dimorphic profile that was inverted by PRS (latency to open arm, *group × sex effect*, *F*_(1,35)_ = 19.184, *p* = 0.0001; % time spent in the open arm, *F*_(1,35)_ = 13.821, *p* = 0.0007; *n* = 8–12 rats/group). Unstressed (control) female rats showed an increased latency to enter the open arm compared to control males (Fisher, #*p* = 0.015). In contrast, PRS females showed reduced latency and increased time spent in the open arm compared to both PRS males (###*p* = 0.00088) and control females (**p = 0.0056). The opposite was found in males, in which PRS increased the latency (**p = 0.0025) and reduced the time spent (*p = 0.049) in the open arm compared to controls. Thus, PRS reduced risk-taking behavior in aged males but caused the opposite effect in females*.*

The study of *spatial recognition memory* in the Y-maze test showed a clear sex-dependent effect (*group × sex effect*, *F*_(1,29)_ = 4.641, *p* = 0.04; *n* = 8–9 rats/group). PRS reduced the recognition score in aged male (23.24% recognition score) (Fisher, *p = 0.014) but not in aged female rats (40.71%) as compared to control rats (43.93% and 37.04% in control males and females, respectively). There was a significant difference between PRS male and PRS female rats (Fischer, #p=0.040) (Fig. 2b).

To examine fine motor skills, we used the *ladder rung-walking test* (Fig. 2c). PRS increased the percentage of errors in both sexes *(group effect*, *F*_(1,35)_=5.235, *p = 0.028, *n* = 8–12 rats/group), but males displayed a higher percentage of errors than females (*sex effect*: *F*_(1,35)_ = 4.55, #*p* = 0.04).

*Exploratory behavior* was assessed in three different tests (Fig. 2d). In the EPM, PRS reduced the number of entries in the closed arm in both sexes (*group effect*, *F*_(1,35)_ = 5.147, *p = 0.04, *n* = 8–12 rats/group). In the open-field test, PRS reduced the distance traveled in both sexes (*group effect*, *F*_(1,36)_ = 11.004, **p = 0.002; *n* = 8–11 rats/group). Furthermore, females showed a greater distance traveled than males (*sex effect*, *F*_(1,36)_ = 20.823, ###*p* = 0.00005). The same profile was observed in the Y-maze, where PRS decreased the total arm entries (*group effect*, *F*_(1,29)_ = 11.023, **p = 0.0024; *n* = 8–9 rats/group), and females showed increased total arm entries compared to males (*sex effect*, *F*_(1,29)_ = 4.251, #*p* = 0.048). Hence, PRS reduced exploratory behavior in both sexes in all three tests.

The bodyweight of rats (Fig. 2e) was not modified by PRS. However, we observed that female rats weigh less than male rats (*sex effect*, *F*_(1,36)_ = 79.565, ###*p* = 0.000001, *n* = 8–12 rats/group).

### Effect of sex and PRS on glutamatergic synapses, synaptic vesicle-associated proteins, and stress-/anti-stress-related proteins in the ventral hippocampus of aged rats

We assessed proteins related to glutamatergic synapses, including metabotropic and ionotropic glutamate receptors and glutamate transporters, synaptic vesicle-associated proteins, and stress-/anti-stress-related proteins in stress-related brain regions (ventral and dorsal hippocampus and prefrontal cortex) and the striatum region.

**Fig. 3:**
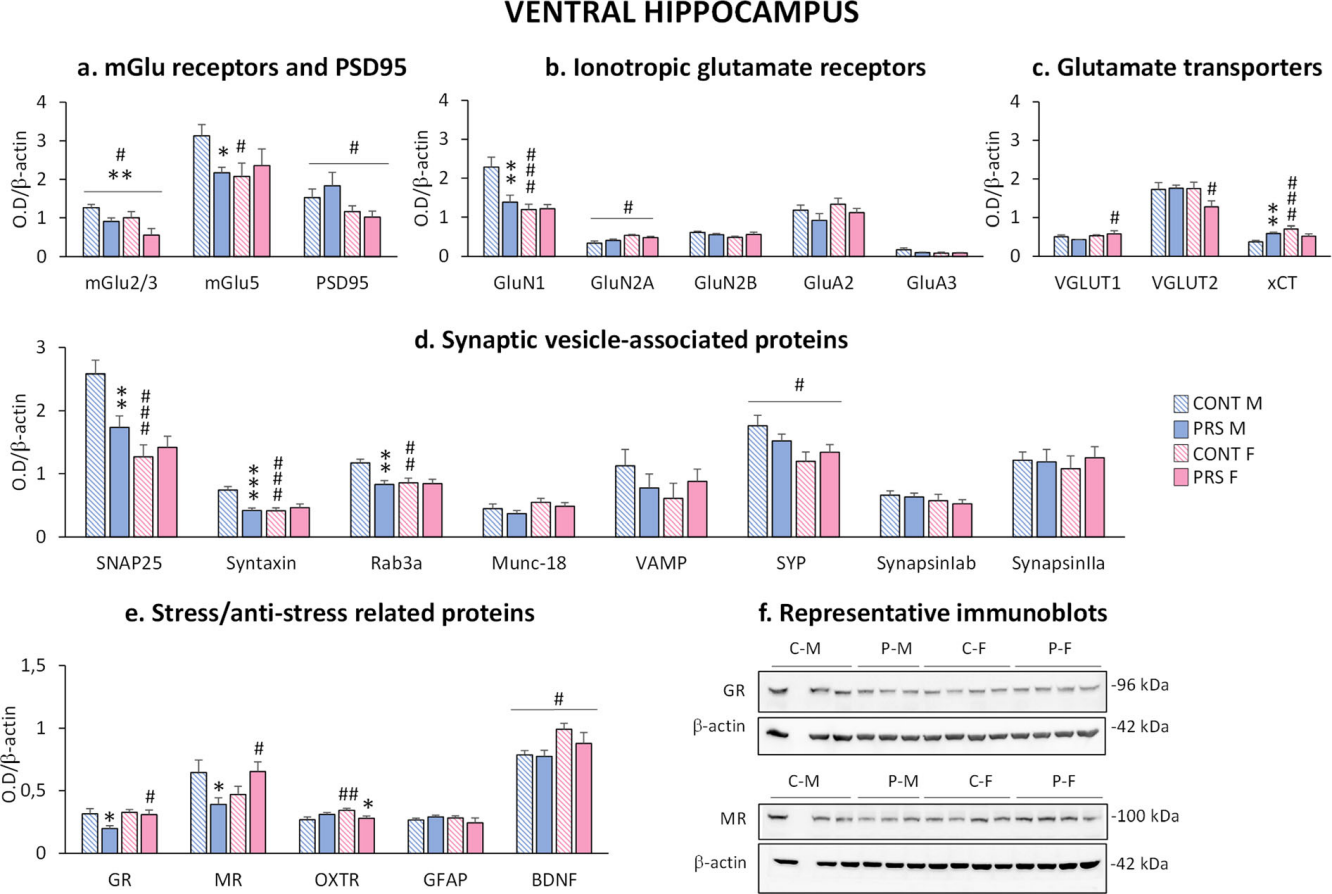
Effect of sex and PRS on glutamatergic synapses, synaptic vesicle-associated proteins and stress-/anti-stress-related proteins in the ventral hippocampus of aged rats. Immunoblot analysis of the biochemical markers of glutamatergic synapses (**a**, **b**, **c**) and synaptic vesicle-associated proteins (**d**) in synaptosomal fractions collected from the ventral hippocampus of aged male and female PRS and control (CONT) rats. Immunoblot analysis of stress-/anti-stress-related proteins in total homogenates (**e**). Representative immunoblots of GR and MR (**f**). Error bars represent the SEM images. CONT vs PRS * = *p* < 0.05; ** = *p* < 0.01; *** = *p* < 0.001. Males vs females # = *p* < 0.05; ## = *p* < 0.01; ### = *p* < 0.001

In the *ventral hippocampus*, PRS decreased mGlu2/3 receptor protein levels in both sexes (*group effect*, *F*_(1,17)_ = 8.472, **p = 0.0097; *n* = 5-6 rats/group) (Fig. 3a). In addition, females, independently of the group, showed reduced mGlu2/3 receptor protein levels compared to males (*sex effect*, *F*_(1,17)_ = 4.827, #*p* = 0.042). PRS reduced mGlu5 receptors (*group × sex effect*, *F*_(1,23)_ = 3.734, *p* = 0.063; *n* = 6–7 rats/group) in males compared to control males (Fisher, *p = 0.049). In addition, control females showed reduced mGlu5 receptors compared to control males (Fisher, #*p* = 0.034). Furthermore, females showed a reduction in postsynaptic density protein 95 (PSD95) (*sex effect*, *F*_(1,19)_ = 6.919, #*p* = 0.016; *n* = 5–7 rats/group) (Fig 3a). The GluN1 subunit of NMDA receptors was changed between groups (*group × sex effect*, *F*_(1,23)_ = 7.362, *p* = 0.012, *n* = 6–7 rats/group). The GluN1 was reduced in PRS males (Fisher, **p = 0.0013) and control females (Fisher, ###*p* = 0.00018), as compared to control males. In contrast, GluN2A protein levels were increased in females compared to males (*sex effect*, *F*_(1,17)_ = 6.944, #*p* = 0.017; *n* = 2–8 rats/group) (Fig 3b).

Interestingly, female PRS rats showed an opposite profile of VGLUT1 and VGLUT2 expression (Fig 3c), with VGLUT1 (*n* = 3–4 rats/group) being increased (Fisher, #*p* = 0.04) and VGLUT2 (*n* = 5–8 rats/group) decreased (Fisher, #*p* = 0.028) in PRS females compared to PRS males. *xCT* protein levels showed significant changes in the ventral hippocampus (*group × sex effect*, *F*_(1,22)_ = 12.394, *p* = 0.0019, *n* = 5–7 rats/group) and were increased in PRS males (Fisher, **p = 0.0026) and control females (Fisher, ###*p* = 0.0008) compared to control males.

For the synaptic vesicle-associated proteins (Fig 3d), we observed a *group × sex interaction* for SNAP25 (*F*_(1,23)_ = 6.879, *p* = 0.015, *n* = 6–7 rats/group), syntaxin (*F*_(1,23)_ = 14.301, *p* = 0.00096, *n* = 6–7 rats/group), and Rab3a (*F*_(1,23)_ = 5.981, *p* = 0.022, *n*= 6–7 rats/group) protein levels. PRS decreased the expression of SNAP25 (Fisher, **p = 0.0051), syntaxin (Fisher, ***p = 0.00014), and Rab3a (Fisher, **p = 0.0018), specifically in males. Moreover, we found lower levels of SNAP25 (Fisher, ###*p* = 0.00007), syntaxin (Fisher, ###*p* = 0.00012), and Rab3a (Fisher, ##*p* = 0.0034) protein levels in control females compared to control males. In addition, synaptophysin (SYP) protein levels were reduced in females compared to males (*sex effect*, *F*_(1,22)_ = 7.73, *p* = 0.011, *n* = 6–7 rats/group).

Stress-/anti-stress-related proteins were modified by PRS and sex (Fig 3e). GR protein levels showed changes (*group × sex effect*, *F*_(1,23)_ = 2.913, *p* = 0.101, *n* = 6–7 rats/group) and were reduced by PRS only in males (Fisher, *p = 0.014). The same profile was observed for MR (*group × sex effect*, *F*_(1,22)_ = 7.703, *p* = 0.011, *n* = 5–7 rats/group), where PRS males showed a reduction in MR compared to control males (Fisher, *p = 0.021). Interestingly, PRS females showed higher GR (Fisher, #*p* = 0.025) and MR (Fisher, #*p* = 0.031) protein levels than PRS males. *OXTR* protein levels were also affected (*group × sex effect*, *F*_(1,23)_ = 8.325, *p* = 0.0083, *n* = 6–7 rats/group) and were higher in control females than in control males (Fisher, ##*p* = 0.007), but PRS reduced OXTR expression in females (Fisher, *p = 0.02). BDNF levels were increased in females, independent of the group (*sex effect*, *F*_(1,22)_ = 6.354, #*p* = 0.019; *n* = 6–7 rats/group). Representative immunoblots of GR and MR are shown in Fig. 3f. All immunoblots are shown in Supplementary Figs. 1, 2, and 3.

### Effect of sex and PRS on glutamatergic synapses, synaptic vesicle-associated proteins, and stress-/anti-stress-related proteins in the dorsal hippocampus of aged rats

**Fig. 4:**
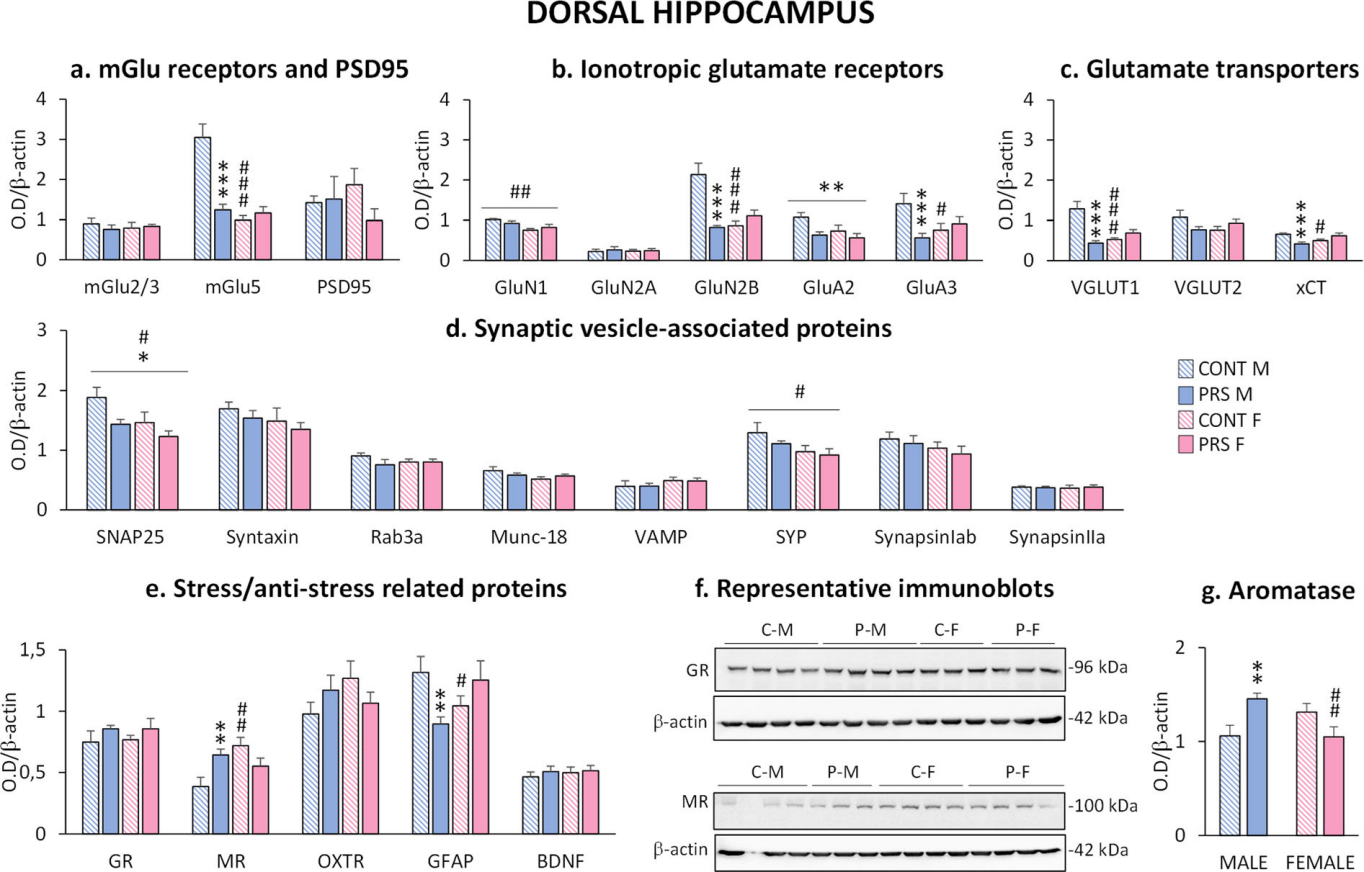
Effect of sex and PRS on glutamatergic synapses, synaptic vesicle-associated proteins and stress-/anti-stress-related proteins in the dorsal hippocampus of aged rats. Immunoblot analysis of the biochemical markers of glutamatergic synapses (**a**, **b**, **c**) and synaptic vesicle-associated proteins (**d**) in synaptosomal fractions collected from the dorsal hippocampus of aged male and female PRS and control (CONT) rats. Immunoblot analysis of the stress-/anti-stress-related proteins in total homogenates (**e**). Representative immunoblots of GR and MR (**f**). Immunoblot analysis of aromatase (**g**). Error bars represent the SEM images. CONT vs PRS * = *p* < 0.05; ** = *p* < 0.01; *** = *p* < 0.001. Males vs females # = *p* < 0.05; ## = *p* < 0.0; ### = *p*<0.001

In the *dorsal hippocampus* (Fig. 4), we found no difference in the mGlu2/3 protein levels, whereas mGlu5 receptors were affected by PRS and sex (*group × sex effect*, *F*_(1,21)_ = 13.886, *p* = 0.0012, *n*= 5–7 rats/group). There was a large reduction in mGlu5 receptor protein levels in PRS males (Fisher, ***p = 0.00003) and control females (Fisher, ###*p* = 0.000001) compared to control males (Fig. 4a). GluN1 protein levels were reduced in females (*sex effect*, *F*_(1,23)_ = 10.308; *p* = 0.0039, *n* = 6–7 rats/group). The GluA2 subunit of AMPA receptors was reduced by PRS in both sexes (*group effect*, *F*_(1,22)_ = 6.179, *p = 0.021; *n* = 5–9 rats/group). We also found a *group × sex interaction* for GluN2B (*group × sex effect*, *F*_(1,21)_ = 11.696, *p* = 0.0026, *n* = 4–9 rats/group), GluA3 (*group × sex effect*, *F*_(1,22)_ = 4.964, *p* = 0.036, *n* = five to nine rats/group), VGLUT1 (*group × sex effect*, *F*_(1,21)_ = 10.885, *p* = 0.0034, *n* = 4–9 rats/group), and xCT (*group × sex effect*, *F*_(1,21)_ = 16.044, *p* = 0.00064, *n* = 5–7 rats/group). The post hoc analysis revealed that PRS males displayed reductions in protein levels compared to control males for GluN2B (Fisher, ***p = 0.00023), GluA3 (Fisher, **p = 0.0091), VGLUT1 (Fisher, ***p = 0.00024), and xCT (Fisher, ***p = 0.00084). Similarly, control females showed a reduction in GluN2B (Fisher, ###*p* = 0.00022), GluA3 (Fisher, #*p* = 0.037), VGLUT1 (Fisher, ###*p* = 0.00082), and xCT (Fisher, #*p* = 0.014) compared to control males (Fig. 4b, 4c).

PRS reduced SNAP25 protein levels in both sexes (*group effect*, *F*_(1,23)_ = 6.487, *p = 0.018; *n* = 6–7 rats/group). In addition, SNAP25 (*sex effect*, *F*_(1,23)_ = 5.484, #*p* = 0.02) and SYP (*sex effect*, *F*_(1,23)_ = 4.956, #*p* = 0.036; *n* = 6–7 rats/group) protein levels were reduced in females of both groups (Fig. 4d).

MR protein levels were altered (*group × sex effect*, *F*_(1,23)_ = 10.853, *p* = 0.0032, *n* = 6–7 rats/group) and were higher in PRS males (Fisher, **p = 0.008) and control females (Fisher, ##*p* = 0.0011) than in control males. A sex-dimorphic profile was observed for GFAP (*group × sex effect*, *F*_(1,22)_ = 8.690, *p* = 0.0074, *n* = 5–7 rats/group). Indeed, PRS reduced GFAP expression only in males (Fisher, **p = 0.0081), while GFAP expression was increased in PRS females (Fisher, #*p* = 0.034) compared to PRS males (Fig. 4e**)**. Representative immunoblots of GR and MR are shown in Fig. 4f. All immunoblots are shown in Supplementary Figs. 4, 5, and 6.

Protein levels of aromatase, an enzyme that converts testosterone into estradiol [51], were analyzed in the dorsal hippocampus by western blot (Fig. 4g). We observed a sex-dimorphic profile induced by PRS (*group × sex effect*, *F*_(1,23)_ = 12.071, *p* = 0.0021; *n* = 6–7 rats/group). PRS increased aromatase levels in males compared to control males (Fisher, **p = 0.0062) and reduced levels in PRS females (Fisher, ##*p* = 0.0071) compared to PRS males.

### Effect of sex and PRS on glutamatergic synapses, synaptic vesicle-associated proteins, and stress-/anti-stress-related proteins in the prefrontal cortex of aged rats

**Fig. 5:**
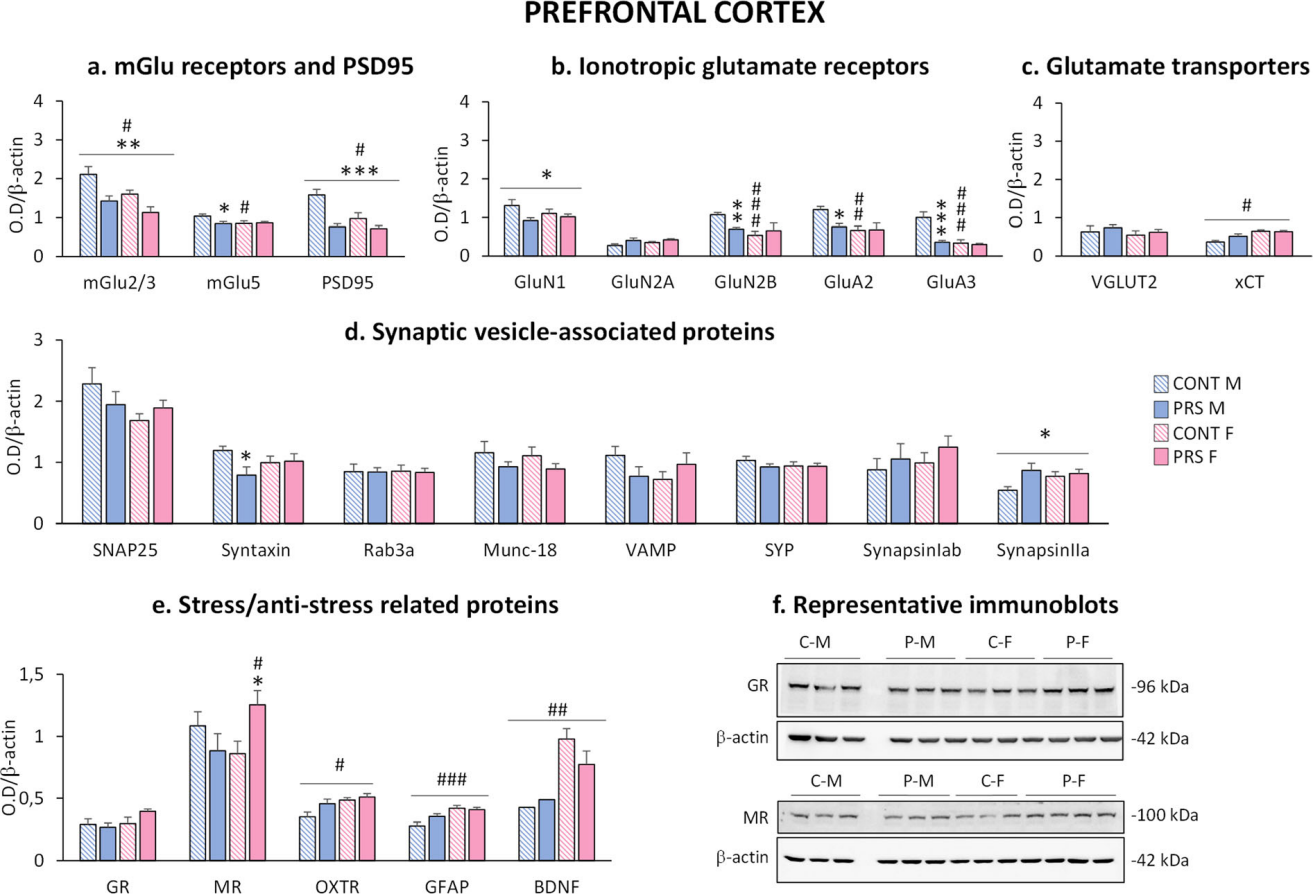
Effect of sex and PRS on glutamatergic synapses, synaptic vesicle-associated proteins and stress-/anti-stress-related proteins in the prefrontal cortex of aged rats. Immunoblot analysis of biochemical markers of glutamatergic synapses (**a**, **b**, **c**) and synaptic vesicle-associated proteins (**d**) in synaptosomal fractions collected from the prefrontal cortex of aged male and female PRS and control (CONT) rats. Immunoblot analysis of the stress-/anti-stress-related proteins in total homogenates (**e**). Representative immunoblots of GR and MR (**f**). Error bars represent the SEM images. CONT vs PRS * = *p* < 0.05; ** = *p* < 0.01; *** = *p* < 0.001. Males vs females # = *p* < 0.05; ## = *p* < 0.01; ### = *p* < 0.001

In the *prefrontal cortex* (Fig. 5), we observed a reduction in mGlu2/3 receptor protein levels in response to PRS (*group effect*, *F*_(1,22)_ = 13.858, **p = 0.0011, *n* = 6–7 rats/group) and in female rats (*sex effect*, *F*_(1,22)_ = 7.436, #*p* = 0.012, *n* = 6–7 rats/group). mGlu5 receptors were also reduced (*group × sex effect*, *F*_(1,22)_ = 3.093, *p* = 0.093, *n* = 6–7 rats/group). In particular, we found a reduction in PRS males (Fisher, *p = 0.02) and control females (Fisher, #*p* = 0.035) compared to control males. Moreover, PSD95 protein levels were decreased by PRS (*group effect*, *F*_(1,22)_ = 25.134, ***p = 0.00005; *n* = 6–7 rats/group) and in female rats (*sex effect*, *F*_(1,22)_ = 5.47, #*p* = 0.029; *n* = 6–7 rats/group) (Fig. 5a). PRS also reduced GluN1 protein levels in both sexes (*group effect*, *F*_(1,22)_ = 5.615, *p = 0.027; *n* = 6–7 rats/group). A *group × sex interaction* was found for (GluN2B (*F*_(1,18)_ = 6.658, *p* = 0.019, *n* = 4–7 rats/group), GluA2 (*F*_(1,20)_ = 3.494, *p* = 0.076; *n* = 6–7 rats/group), and GluA3 (*F*(_1,22)_ = 11.528, *p* = 0.0026; *n* = 5–7 rats/group). PRS males displayed a reduction in protein expression compared to control males for GluN2B (Fisher, **p = 0.0045), GluA2 (Fisher, *p = 0.016), and GluA3 (Fisher, ***p = 0.00001). Similarly, control females showed reduced GluN2B (Fisher, ###*p* = 0.00096), GluA2 (Fisher, ##*p* = 0.0059), and GluA3 (Fisher, ###*p* = 0.00004) protein levels, compared to control males (Fig. 5b). Furthermore, females of both groups showed an increase in xCT protein levels (*sex effect*, *F*_(1,19)_ = 5.76, #*p* = 0.027; *n* = 4–7 rats/group) (Fig. 5c).

Few changes in the synaptic vesicle-associated with the prefrontal cortex were observed (Fig. 5d). Syntaxin expression was changed (*group × sex effect*, *F*_(1,23)_ = 14.301, *p* = 0.067, *n* = 6–7 rats/group) and reduced in PRS males compared to control males (Fisher, *p = 0.015). In contrast, synapsin IIa expression was increased by PRS in both sexes (*group effect*, *F*_(1,23)_ = 5.318, *p = 0.011; *n* = 6–7 rats/group).

MR protein levels were altered (*group × sex effect*, *F*_(1,20)_ = 6.486, *p* = 0.019, *n* = 6 rats/group) and were increased in PRS females compared to PRS males (Fisher, *p = 0.036) and control females (Fisher, #*p* = 0.028). Furthermore, females of both groups displayed greater OXTR protein levels (*sex effect*, *F*_(1,19)_ = 8.829, #*p* = 0.01; *n* = 5–6 rats/group). Interestingly, we found a higher expression of BDNF (*sex effect*, *F*_(1,20)_ = 13.705, ##*p* = 0.0014; *n* = 6 rats/group) and GFAP (sex effect, *F*_(1,19)_ = 16.466, ###*p* = 0.00067; *n* = 6 rats/group) in females of both groups (Fig. 5e). Representative immunoblots of GR and MR are shown in Fig. 5f. All immunoblots are shown in Supplementary Figs. 7, 8, and 9.

### Effect of sex and PRS on glutamatergic synapses and synaptic vesicle-associated proteins in the striatum of aged rats

**Fig. 6:**
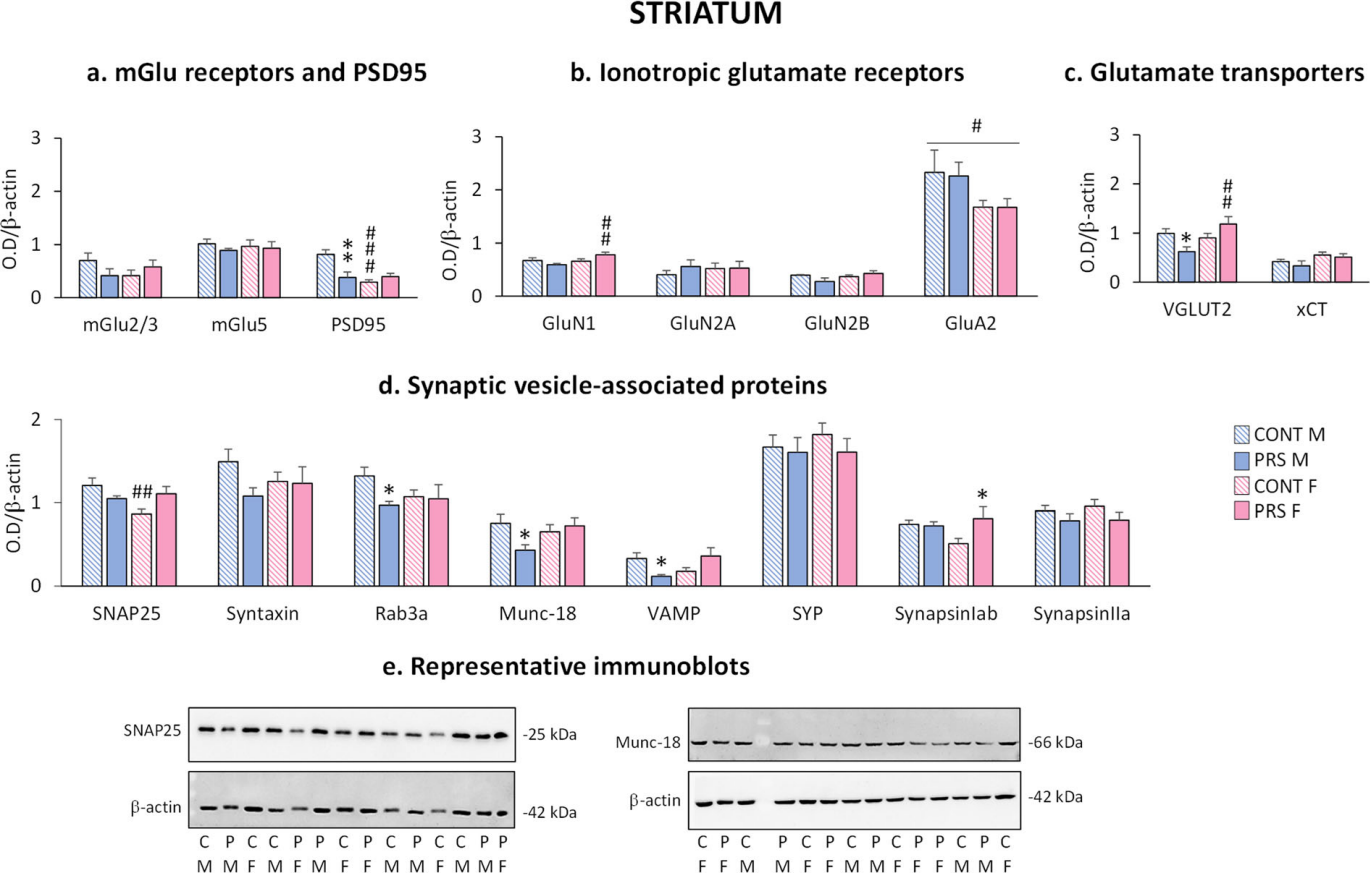
Effect of sex and PRS on glutamatergic synapses and synaptic vesicle-associated proteins in the striatum of aged rats. Immunoblot analysis of biochemical markers of glutamatergic synapses (**a**, **b**, **c**) and synaptic vesicle-associated proteins (**d**) in synaptosomal fractions collected from the striatum of aged male and female PRS and control (CONT) rats. Representative immunoblots of SNAP-25 and Munc-18 (**e**). Error bars represent the SEM images. CONT vs PRS * = *p* < 0.05; ** = *p* < 0.01; males vs females # = *p* < 0.05; ## = *p* < 0.01; ### = *p* < 0.001

In the *striatum* (Fig. 6), PSD95 protein levels were reduced (*group × sex effect*, *F*_(1,18)_ = 11.978, *p* = 0.0028; *n* = 4–7 rats/group) in PRS males (Fisher, **p = 0.0013) and control females (Fisher, ###*p* = 0.00009) compared to control males (Fig. 6a). Moreover, PRS increased GluN1 (*group × sex effect*, *F*_(1,23)_ = 5.24, *p* = 0.032, *n* = 6–7 rats/group) only in females (Fisher, ##p = 0.0056). GluA2 was decreased in females of both groups (*sex effect*: *F*_(1,18)_ = 4.872, #*p* = 0.04; *n* = 4–6 rats/group) (Fig. 6b). Concerning VGLUT2 protein levels, we found a sex-dimorphic profile, which was inverted by PRS (*group × sex effect*, *F*_(3,18)_ = 9.119, *p* = 0.0074; *n* = 4–7 rats/group). PRS decreased VGLUT2 in males (Fisher, *p = 0.035) but increased VGLUT2 in females (Fisher, ##*p* = 0.0036) compared to control male and PRS male rats, respectively (Fig. 6c).

Synaptic vesicle-associated proteins were modified by sex and PRS (Fig. 6d). We observed a *group × sex interaction* for the expression of SNAP25 (*F*_(1,23)_ = 8.441, *p* = 0.008; *n* = 6–7 rats/group), Munc-18 (*F*_(1,23)_ = 4.26, *p* = 0.05; *n* = 5–7 rats/group), and VAMP (*F*_(1,21)_ = 11.51, *p* = 0.0027; *n* = 5–7 rats/group). PRS males displayed a reduction in Rab3a (Fisher, *p = 0.016), Munc-18 (Fisher, *p = 0.017), and VAMP (Fisher, *p = 0.015) protein levels compared to control males. Moreover, control females showed reduced SNAP25 protein levels (Fisher, ##*p* = 0.0022). Similarly, protein levels of synapsin Iab were modified (*group × sex effect*, *F*_(1,24)_ = 11.51, *p* = 0.074; *n* = 7 rats/group) and were increased in PRS females (Fisher, *p = 0.02) compared to control females. Representative immunoblots of SNAP25 and Munc-18 are shown in Fig. 6f. All immunoblots are shown in Supplementary Figs. 10 and 11.

### Multidimensional analysis

Consecutively with the western blot analysis, we performed a multidimensional analysis with all the protein data obtained in each structure to analyze the effect of PRS and sex on the protein data set more comprehensively.

**Fig. 7:**
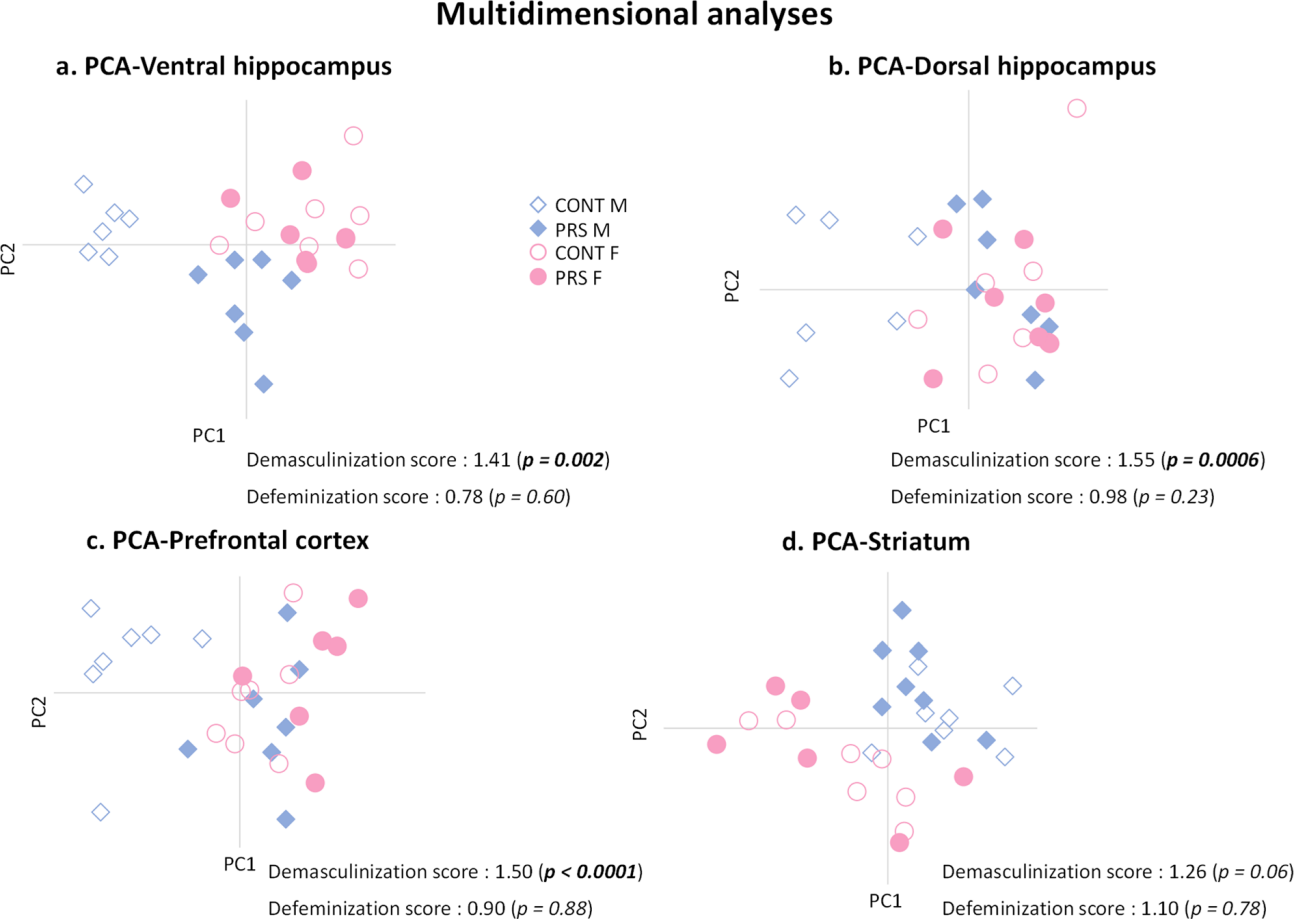
Multidimensional analyses. Multidimensional analysis of the protein data set (markers of the glutamatergic synapse, synaptic vesicles-associated proteins, and stress-/anti-stress-related proteins) of the ventral hippocampus (**a**), dorsal hippocampus (**b**), prefrontal cortex (**c**), and striatum (**d**). PCA, principal component analysis

The multidimensional analyses revealed a demasculinization profile of the glutamatergic synapse induced by PRS in the ventral hippocampus (Fig. 7a; *demasculinization score*, 1.41; Mann–Whitney–Wilcoxon test, *p* = 0.002; *n* = 5–7 rats/group), in the dorsal hippocampus (Fig. 7b; *demasculinization score*, 1.55; Mann–Whitney–Wilcoxon test, *p* = 0.0006; *n* = 5-9 rats/group) as well as in the prefrontal cortex (Fig. 7c; *demasculinization score*, 1.50; Mann–Whitney–Wilcoxon test, *p* < 0.0001; *n* = 6–7 rats/group) but not in the striatum (Fig. 7d; *demasculinization score*, 1.26; Mann–Whitney–Wilcoxon test, *p* = 0.06; *n* = 6–7 rats/group). PRS did not induce a global defeminization profile of glutamatergic synapses in any of the brain regions studied. Of note, multidimensional analyses performed on the stress-/anti-stress-related protein data set revealed that PRS induced a defeminization profile only in the ventral hippocampus (*defeminization score*, 1.71; Mann–Whitney–Wilcoxon test, *p* = 0.004; *n* = 5–7 rats/group) (Supplementary Fig. 12a) and a demasculinization profile exclusively in the dorsal hippocampus (*demasculinization score*, 1.73; Mann–Whitney–Wilcoxon test, *p* = 0.001; *n* = 5–9 rats/group) (Supplementary Fig. 12b). Thus, demasculinization could be observed only for proteins related to glutamatergic transmission.

### Effect of sex and PRS on sex hormones and peripheral markers in aged male and female rats

**Fig. 8:**
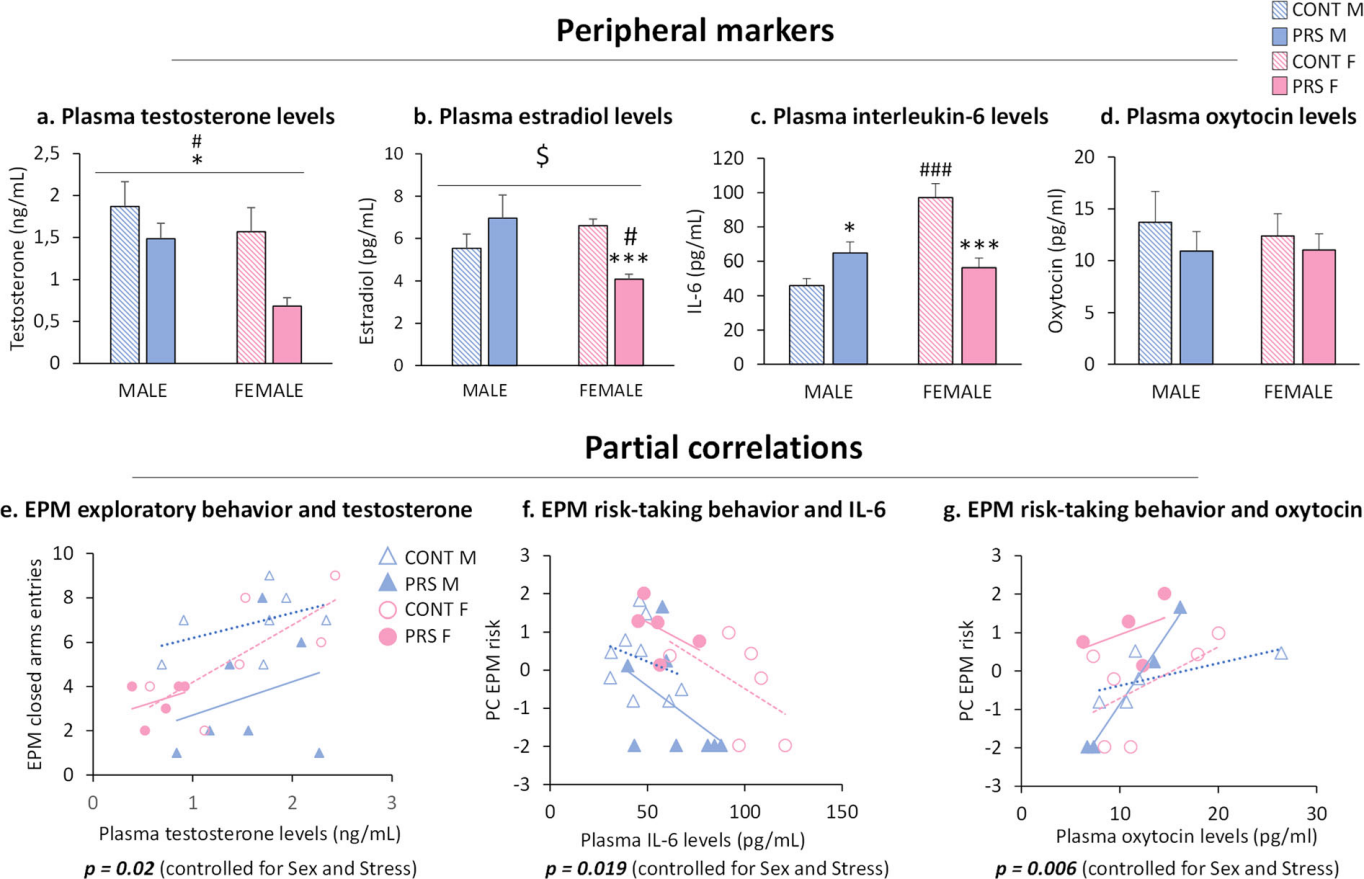
Effect of sex and PRS on sex hormones and peripheral markers in aged male and female rats. Plasma levels of testosterone (ng/mL) (**a**), estradiol (pg/mL) (**b**), interleukin-6 (pg/mL) (**c**), and oxytocin (pg/mL) (**d**). Partial correlation between exploratory behavior in the EPM and testosterone levels is shown in **e**. Partial correlation between risk-taking behavior and IL-6 is represented in **f**. Partial correlation between risk-taking behavior and oxytocin levels is shown in **g**. Error bars represent the SEM. CONT vs PRS * = *p* < 0.05; *** = *p* < 0.001. Males vs females # = *p* < 0.05; ### = *p* < 0.001

We assessed plasma testosterone and estradiol levels (Fig. 8a, 8b). We found a significant reduction in testosterone levels in response to PRS in both sexes (*group effect*, *F*_(1,24)_ = 5.882, *p = 0.023; *n*= 5–9 rats/group). Moreover, females displayed lower levels of testosterone than males (*sex effect*, *F*_(1,24)_ = 4.42, #*p* = 0.046; *n* = 5–9 rats/group) (Fig. 8a). Estradiol levels in the plasma revealed a *group × sex interaction* (*F*_(1, 21)_ = 5.3, $*p* = 0.032; *n* = 4–8 rats/group) where PRS females showed lower levels of estradiol than control females (one-way ANOVA, ***p = 0.00021) and PRS males (one-way ANOVA, #*p* = 0.034) (Fig. 8b).

We also measured plasma levels of IL-6 and oxytocin as peripheral markers of inflammation and resilience to stress, respectively (Fig. 8c, 8d). We found a group × sex interaction for IL-6 levels (*group × sex effect*, *F*_(1,20)_ = 22.901, *p* = 0.00007; *n* = 5–9 rats/group). PRS largely reduced IL-6 levels in females (Fisher, ***p = 0.00031) but not males, which displayed higher IL-6 levels than control males (Fisher, *p = 0.024). Furthermore, control females showed an increase in IL-6 levels (Fisher, ###*p* = 0.000003) compared to control males. We did not observe changes in oxytocin levels by PRS (*F*_(1,15)_ = 0.657; *p = 0.43*; *n* = 4–6 rats/group) or sex (*F*_(1,15)_ = 0.059; *p* = 0.81*).*

We performed *partial correlations* to examine the relationships between behavior and sex hormones and two peripheral markers (IL-6 and oxytocin) in relation to PRS and sex. We found a positive correlation between exploratory behavior and testosterone levels (*p* = 0.02; *n* = 5–7 rats/group). In addition, we observed a negative correlation between risk-taking behavior in the EPM and levels of IL-6 (*p* = 0.019; *n* = 5–9 rats/group; Fig. 8f) and a positive correlation with oxytocin levels (*p* = 0.006; *n* = 4–6 rats/group; Fig. 8g).

## Discussion

We showed that perinatal stress programs lifelong changes in mechanisms that help to balance vulnerability and resilience to stress. Indeed, non-genetic factors such as stress occurring early in life are known to act as perinatal “programming” because they critically contribute to several aspects of the adult phenotype. This concept is based on Barker’s theory of the evolutionary origins of adult health and disease (DOHaD). This theory defines the relationship between early developmental influences on health and disease in adulthood and aging [52–54]. We observed that long-term programming was sex-dependent in both non-stressed and PRS aged rats. At a behavioral level, PRS increased risk-taking behavior in females but decreased it in males. These results are similar to those observed in adult (3–6 month old) rats [32], indicating that the sex-dependent programming induced by PRS is persistent and uniform. While PRS impaired spatial memory in males, no changes were observed in females. These results are also consistent with previous findings obtained in males [16] and suggest that females are resilient to maladaptive neuroplasticity changes caused by early-life stress. Motor performance declines with aging, as shown by increased variability of movement [55], slowing of movement [56], altered coordination [57], and difficulties with balance and gait [58]. Using the ladder rung-walking test, we showed that PRS increased the number of errors predominantly in males, lending credit to the hypothesis that aged females cope better with PRS, even if PRS decreased exploratory behavior in the EPM, Y-maze, and open-field tests equally in both sexes.

Impressively, these alterations were associated with large reductions in biochemical markers of the glutamatergic synapse in the hippocampus (ventral and dorsal) and prefrontal cortex, which were exclusively seen in males. PRS decreased both the mGlu5 receptor and GluN1 subunit protein levels in male rats. A similar scenario was reported in adult rats, in which PRS reduced hippocampal mGlu5 receptors only in males [32]. mGlu5 and NMDA receptors are physically linked by a chain of scaffolding proteins and functionally interact in the induction of activity-dependent synaptic plasticity [59, 60]. Changes in mGlu5 and NMDA receptors found in this study are in agreement with previous data observed in adult PRS male rats [61] and suggest that PRS impairs the receptor substrates of synaptic plasticity in aged male rats. Interestingly, the lowering effect of PRS on mGlu2/3 receptor protein levels in the ventral hippocampus and prefrontal cortex of aged rats was not sex-dependent, whereas it was sex-dependent in non-stressed control rats, with control females showing reduced mGlu2/3 protein levels as compared to control males. Again, the PRS effects were similar to those reported in the whole hippocampus of adult rats, where PRS reduced mGlu2/3 receptor protein levels in both sexes [32]. Activation of the mGlu2/3 receptors with the agonist, LY354740, induces increased risk-taking behavior in male rats [62, 63]. mGlu2/3 receptors are targets for drug treatment of anxiety and other stress-related disorders in humans [64]. Here, reduced mGlu2/3 receptor expression was associated with decreased exploratory behavior in PRS rats of both sexes. mGlu2/3 receptors are preferentially localized in presynaptic terminals and are known to negatively regulate glutamate release [65]. Using adult rats, we found that PRS reduces glutamate release in the ventral hippocampus [26]. Hence, the reduction in mGlu2/3 receptor expression seen during early-life [66], adult life [32], and aging (present data) could represent an allostatic compensatory mechanism, which operates across the entire lifespan. One of the most interesting findings related to a possible hypofunction of the glutamatergic synapse in PRS males was the reduction of NMDA and AMPA receptor subunits. NMDA and AMPA receptors are ligand-gated ion channels involved in the induction and expression of long-term potentiation and long-term depression of excitatory synaptic transmission, respectively [67, 68]. In agreement with our results, it has been reported that early-life stress impairs the development of synaptic plasticity in the CA1 hippocampal region in a sex-dependent manner, with males being more vulnerable [69]. Furthermore, a decrease in GluN2B following early-life stress has been reported in the hippocampus [70]. Interestingly, xCT protein levels in PRS male rats differed between the dorsal and ventral hippocampi. xCT protein levels in male rats were increased in the ventral hippocampus and decreased in the dorsal hippocampus in response to PRS. xCT is the catalytic subunit of X_c_^-^, the cysteine glutamate antiporter that supports the endogenous activation of presynaptic mGlu2 receptors by enhancing glutamate efflux from astrocytes [71]. xCT in the hippocampal dentate gyrus has been implicated in the mechanisms of resilience to stress [72], and changes in glutamate homeostasis in response to stress differ between the ventral and dorsal hippocampi [26, 72]. Hence, the differential expression of xCT in the ventral and dorsal hippocampus of PRS rats could help explain the greater involvement of the ventral hippocampus in the “pathological” phenotype triggered by PRS.

Glutamatergic neurotransmission is also regulated by the expression and function of synaptic vesicle-related proteins involved in glutamate release [73, 74]. Synaptic vesicle-associated proteins were decreased by PRS in the ventral hippocampus and striatum of male rats, suggesting that PRS predisposes to long-term dysfunction of the release machinery in aged rats. The hypofunction of glutamate transmission observed in the ventral hippocampus of aged male PRS rats is in agreement with previous results obtained in adult male PRS rats [26, 27, 75]. This supports the hypothesis that long-lasting alterations in the glutamate synapse in the ventral hippocampus lie at the core of the programming triggered by PRS.

Compared to males, females showed reduced body weight regardless of early-life stress. This may have a potential impact on the sex-specific dimorphic profile observed in aged rats. Indeed, it has been shown that caloric restriction and subsequent weight loss during aging could prevent the reprogramming of daily rhythms in mice caused by aging [76]. The reduced body weight in females, associated with increased levels of BDNF and MR, supports neuroprotection. PRS did not induce changes in body weight in both sexes during aging. This was not surprising because we have previously shown that PRS induced caloric restriction and reduced body weight at birth but not in adult life [77]. The recovery of body weight after early postnatal life in PRS rats might reflect adaptive mechanisms driven by growth-related metabolic alterations in these rats [30, 31, 77–79].

Glucocorticoids regulate glutamate transmission via MRs and GRs [22, 80, 81]. A link between MRs and mGlu receptors is suggested by evidence that glucocorticoids, acting via MRs, decrease resilience to stress by downregulating mGlu2 receptors [82]. The activation of GRs by glucocorticoids mediates the negative feedback of the HPA axis. Therefore, decreased GR and MR expression contribute to the long-lasting dysregulation of the HPA axis. Accordingly, prolonged corticosterone secretion following acute stress associated with reduced GR/MR protein levels in the hippocampus has been found in male adult PRS rats [15, 20]. In addition, circulating glucocorticoid levels in PRS middle-aged male **[**16**]** and female [30, 31] rats were similar to those of old non-stressed control rats, suggesting that PRS accelerates the age-related dysfunction of the HPA axis [16, 30, 31, 83, 84]. Here, we showed that PRS decreased GR and MR protein levels in the ventral hippocampus of male rats, whereas MR levels were increased in female rats. This suggests that PRS female rats are more protected than PRS male rats against the accelerated age-related dysfunction of the HPA axis. Furthermore, impairment of the HPA axis in aged PRS rats may be involved in the programming of a long-lasting hypofunction of the glutamatergic synapse ([26]; and present data). Oxytocin acts as an anti-stress hormone [25, 85, 86]) and supports maternal care [46, 86, 87]. Although plasma oxytocin levels were unchanged by PRS in both sexes, we observed increased oxytocin receptors in the prefrontal cortex of female PRS rats, indicating increased oxytocinergic transmission. BDNF is another key factor in the stress response. Like oxytocin, BDNF has anti-stress effects, as shown by evidence that the overexpression of BDNF occludes the effects of chronic stress [88] and chronic stress decreases BDNF levels [89]. BDNF-mediated signaling is involved in the structural effects of stress and plays an important role in dendritic remodeling [90, 91]. In addition, BDNF expression is influenced by maternal separation early in life [92]. GFAP levels in the dorsal hippocampus were increased by PRS in females and reduced in males. GFAP is an astrocyte-specific intermediate filament protein [93, 94], which increases during reactive astrogliosis associated with neurodegeneration.

Multidimensional analyses of the overall data on protein expression showed that PRS in male rats induced a “demasculinization profile” of glutamatergic transmission and synaptic vesicle-related proteins in the ventral and dorsal hippocampus and prefrontal cortex, the main regions related to stress [95], but not in the striatum. In contrast, PRS did not alter the profile in female rats. That is, it did not induce defeminization. These findings are consistent with previous data obtained in adult rats [42]. Thus, PRS demasculinizes rather than defeminizes, and indeed male PRS rats exhibited a profile similar to that of female PRS rats. Indeed, masculinization or feminization allows for the capacity to express sex-specific profiles in adulthood, and demasculinization or defeminization eliminates or reduces the capacity to express sex-specific profiles in adulthood. Demasculinization might reflect changes in sex steroid hormones. Indeed, PRS males showed decreased testosterone and increased estradiol levels in plasma. A reduction in testosterone levels might represent a lifelong endocrine outcome of PRS in male rats because it was observed in fetal and adult life after maternal stress [29, 37, 43]. Thus, the sex hormonal change induced by PRS could be taken into account in relation to the demasculinization of the glutamatergic system observed during old age. The higher GFAP levels found in the prefrontal cortex of aged female rats in both groups were associated with lower testosterone levels compared to male rats. However, this association was not found in the dorsal hippocampus of PRS male rats, where GFAP levels were largely reduced despite the reduction in plasma testosterone levels compared to unstressed male rats. Thus, changes in GFAP expression driven by sex and PRS in aged rats appear to be brain region-dependent and might reflect a complex interaction between peripheral sex steroids and intrinsic brain mechanisms. Interestingly, we found that aromatase, the enzyme that converts testosterone to estradiol, was upregulated by PRS in males and downregulated in females in the dorsal hippocampus but not in the ventral hippocampus or prefrontal cortex (data not shown), supporting the region-specific regulation of GFAP by sex steroids.

Glucocorticoids and the immune system are tightly linked, and alterations in the immune system were found to be induced by PRS in adult life, as shown by a pro-inflammatory state [18]. Here, we found that PRS increased IL-6 levels in males but caused the opposite effect in females. It is known that IL-6 levels increase with age in both rats [96] and humans [97]. Thus, our findings suggest that females are protected against systemic inflammation during aging, which is caused by early-life stress. Interestingly, rats with higher levels of IL-6 showed reduced risk-taking behavior in the EPM test, and this negative correlation is in line with the current belief that inflammation highly contributes to behavioral changes associated with age and age-related disorders. Finally, the levels of the anti-stress hormone, oxytocin, which corrects behavioral and biochemical abnormalities caused by PRS in adult rats [46], showed a positive correlation with risk-taking behavior, supporting the view that oxytocin has a protective effect against the increased vulnerability to stress caused by PRS.

**Fig. 9:**
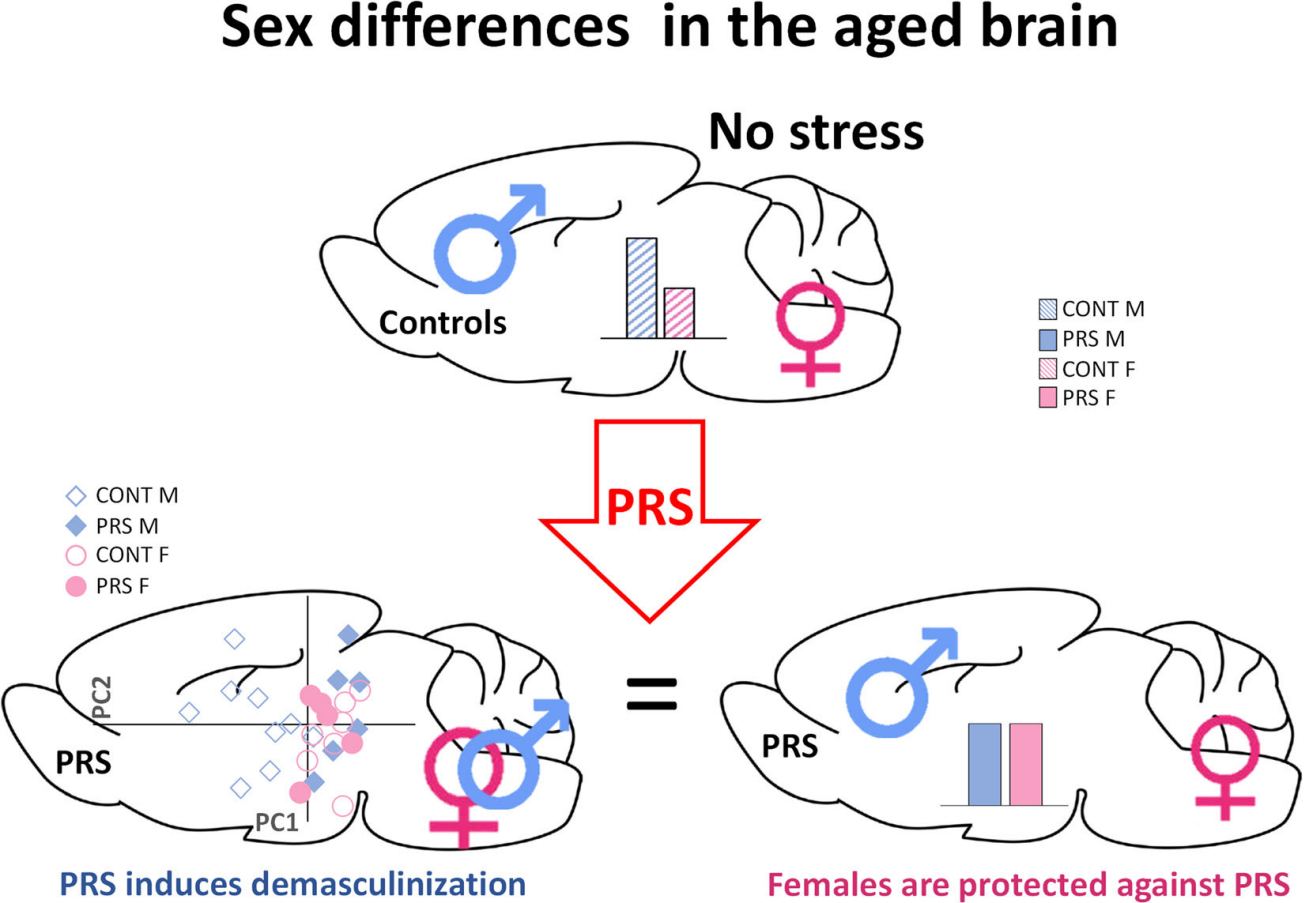
Graphical abstract. Aged male and female control rats display sex differences in the brain. PRS induces brain demasculinization, thereby abolishing sex differences. PRS does not affect females as much as males during aging, suggesting that females are protected against PRS. Males are represented in blue, and females are represented in pink. E = embryologic day; P = postnatal day

In conclusion, our findings provide the first evidence that the effects of PRS are long-term programmed and sex-dependent. PRS induces a demasculinization profile of glutamate and synaptic vesicle-related proteins in aged male rats but not defeminization in female rats. The early-lifelong programming induced by PRS strongly supports Barker’s hypothesis of adult health and disease (DOHaD). In aged PRS females, the lower systemic levels of the pro-inflammatory cytokine, IL-6, and the higher levels of BDNF in stress-related brain regions might be components of an adaptive mechanism aimed at restraining age-dependent neuroinflammation and neurodegeneration. Along this line, demasculinization observed in PRS male rats might reflect an allostatic load reaction against PRS, as hypothesized in Fig. 9. This kind of studies aimed at understanding the mechanisms involved in the programming of aging in both sexes may contribute to identifying early environmental factors and pharmacological treatment strategies involving glutamate transmission. Finally, due to people living longer, and thus, the population of elderly growing worldwide, such studies could help improve older adults’ quality of life.

## Supplementary information

Supplementary Figure 1. Uncropped images of the immunoblots of the glutamatergic synapse markers in the ventral hippocampus (C: control, P: PRS, M: male, F: female). (PNG 754 kb)

High resolution image (TIF 1944 kb)

Supplementary Figure 2. Uncropped images of the immunoblots of the synaptic vesicle-associated proteins in the ventral hippocampus (C: control, P: PRS, M: male, F: female). (PNG 644 kb)

High resolution image (TIF 1740 kb)

Supplementary Figure 3. Uncropped images of the immunoblots of the stress/anti-stress related proteins in the ventral hippocampus (C: control, P: PRS, M: male, F: female). (PNG 338 kb)

High resolution image (TIF 753 kb)

Supplementary Figure 4. Uncropped images of the immunoblots of the glutamatergic synapse markers in the dorsal hippocampus (C: control, P: PRS, M: male, F: female). (PNG 678 kb)

High resolution image (TIF 1631 kb)

Supplementary Figure 5. Uncropped images of the immunoblots of the synaptic vesicle-associated proteins in the dorsal hippocampus (C: control, P: PRS, M: male, F: female). (PNG 503 kb)

High resolution image (TIF 1195 kb)

Supplementary Figure 6. Uncropped images of the immunoblots of the stress/anti-stress related proteins and aromatase in the dorsal hippocampus (C: control, P: PRS, M: male, F: female). (PNG 348 kb)

High resolution image (TIF 764 kb)

Supplementary Figure 7. Uncropped images of the immunoblots of the glutamatergic synapse markers in the prefrontal cortex (C: control, P: PRS, M: male, F: female). (PNG 687 kb)

High resolution image (TIF 1714 kb)

Supplementary Figure 8. Uncropped images of the immunoblots of the synaptic vesicle-associated proteins in the prefrontal cortex (C: control, P: PRS, M: male, F: female). (PNG 600 kb)

High resolution image (TIF 1501 kb)

Supplementary Figure 9. Uncropped images of the immunoblots of the stress/anti-stress related proteins and aromatase in the prefrontal cortex (C: control, P: PRS, M: male, F: female). (PNG 297 kb)

High resolution image (TIF 660 kb)

Supplementary Figure 10. Uncropped images of the immunoblots of the glutamatergic synapse markers in the striatum (C: control, P: PRS, M: male, F: female). (PNG 564 kb)

High resolution image (TIF 1400 kb)

Supplementary Figure 11. Uncropped images of the immunoblots of the synaptic vesicle-associated proteins in the striatum (C: control, P: PRS, M: male, F: female). (PNG 561 kb)

High resolution image (TIF 1358 kb)

Supplementary Figure 12. Multidimensional analyses of the stress/anti-stress related proteins in the ventral hippocampus (**a**), dorsal hippocampus (**b**), and prefrontal cortex (**c**). PCA: principal component analysis. (PNG 100 kb)

High resolution image (TIF 227 kb)

## Abbreviations

AMPA: α-Amino-3-hydroxy-5-méthylisoazol-4-propionate acid
BDNF: Brain-derived neurotrophic factor
C: Control
CNS: Central nervous system
CONT: Control
COVID-19: Coronavirus disease 2019
DOHaD: Developmental Origins of Health and Disease
E_2_: Estradiol
EPM: Elevated plus maze
F: Female
GFAP: Glial fibrillary acidic protein
GR: Glucocorticoid receptor
HPA: Hypothalamic–pituitary–adrenal axis
IL-6: Interleukin-6
ITI: Intertrial interval
M: Male
mGlu: Metabotropic glutamate receptor
MR: Mineralocorticoid receptor
NMDA: N-Methyl-D-aspartate
OXTR: Oxytocin receptor
P: Perinatal stress
PC: Principal component
PCA: Principal component analysis
PERMANOVA: Permutational MANOVA
PRS: Perinatal stress
PSD95: Postsynaptic density protein 95
SNAP-25: Synaptosomal-associated protein, 25 kDa
SYP: Synaptophysin
VAMP: Vesicle-associated membrane proteins
vGLUT1: Vesicular glutamate transporter 1
VGLUT2: Vesicular glutamate transporter 2
xCT: Cystine/glutamate antiporter
