# Supplementary figures and images for: Maternal stress programs a demasculinization of glutamatergic transmission in stress-related brain regions of aged rats
Source: GeroScience. 2021 May 13;44(2):1047–69. doi: 10.1007/s11357-021-00375-5 (PMC8116647; doi:10.1007/s11357-021-00375-5)

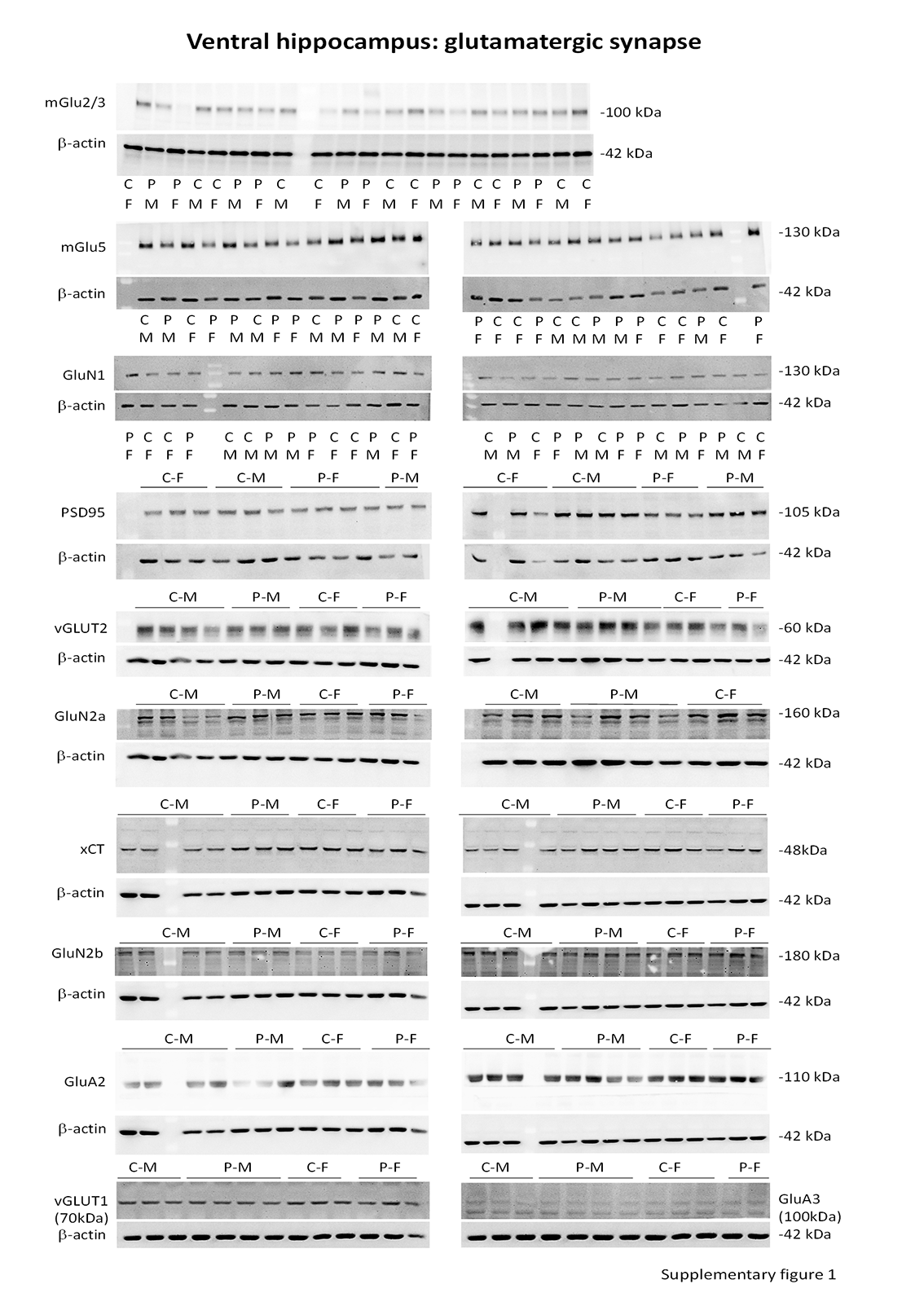

Supplement: Supplementary file 1 — Uncropped images of the immunoblots of the glutamatergic synapse markers in the ventral hippocampus (C: control, P: PRS, M: male, F: female). (PNG 754 kb) [file 11357_2021_375_Fig10_ESM.png]

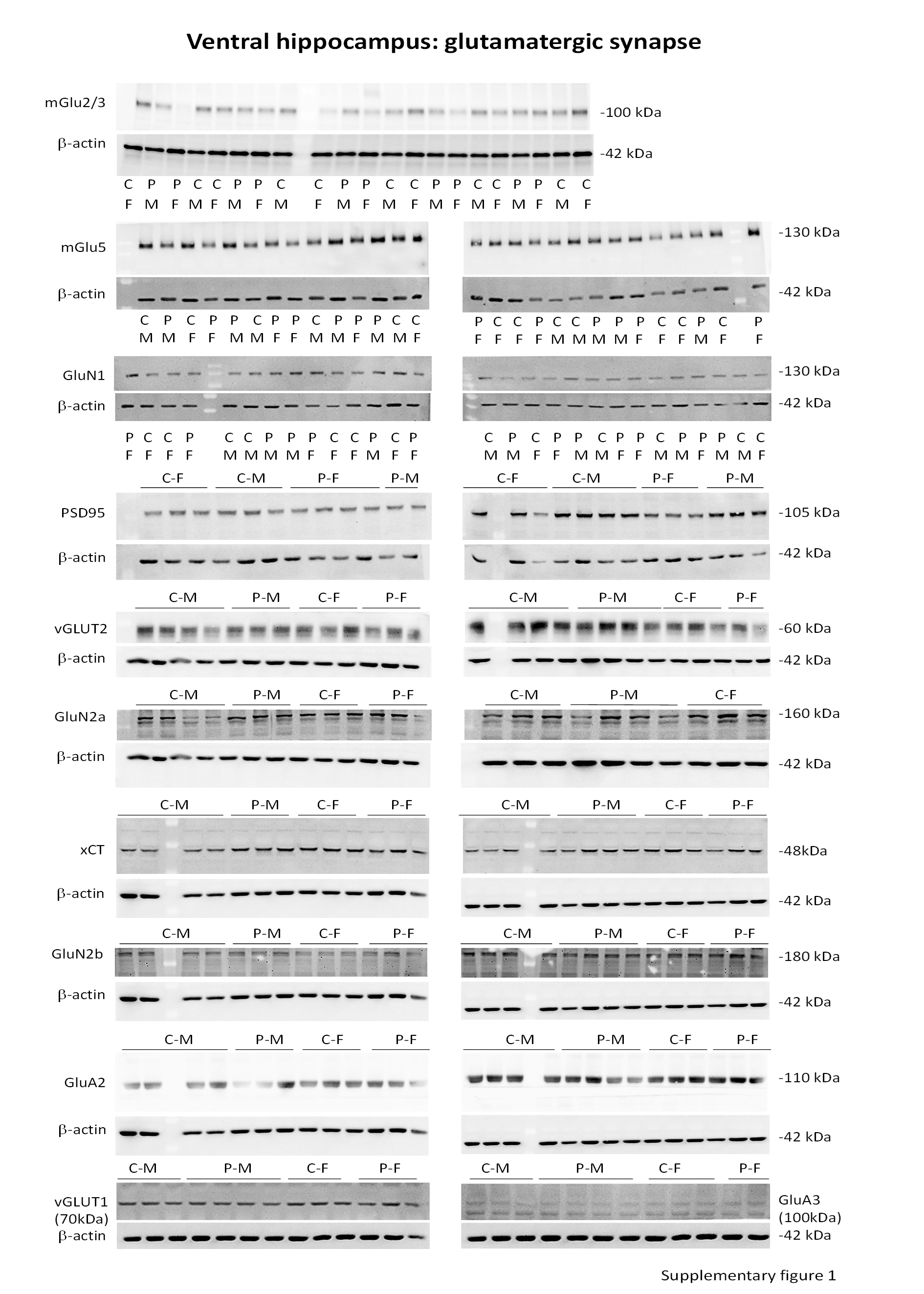

Supplement: Supplementary file 2 — High resolution image (TIF 1944 kb) [file 11357_2021_375_MOESM1_ESM.tif]

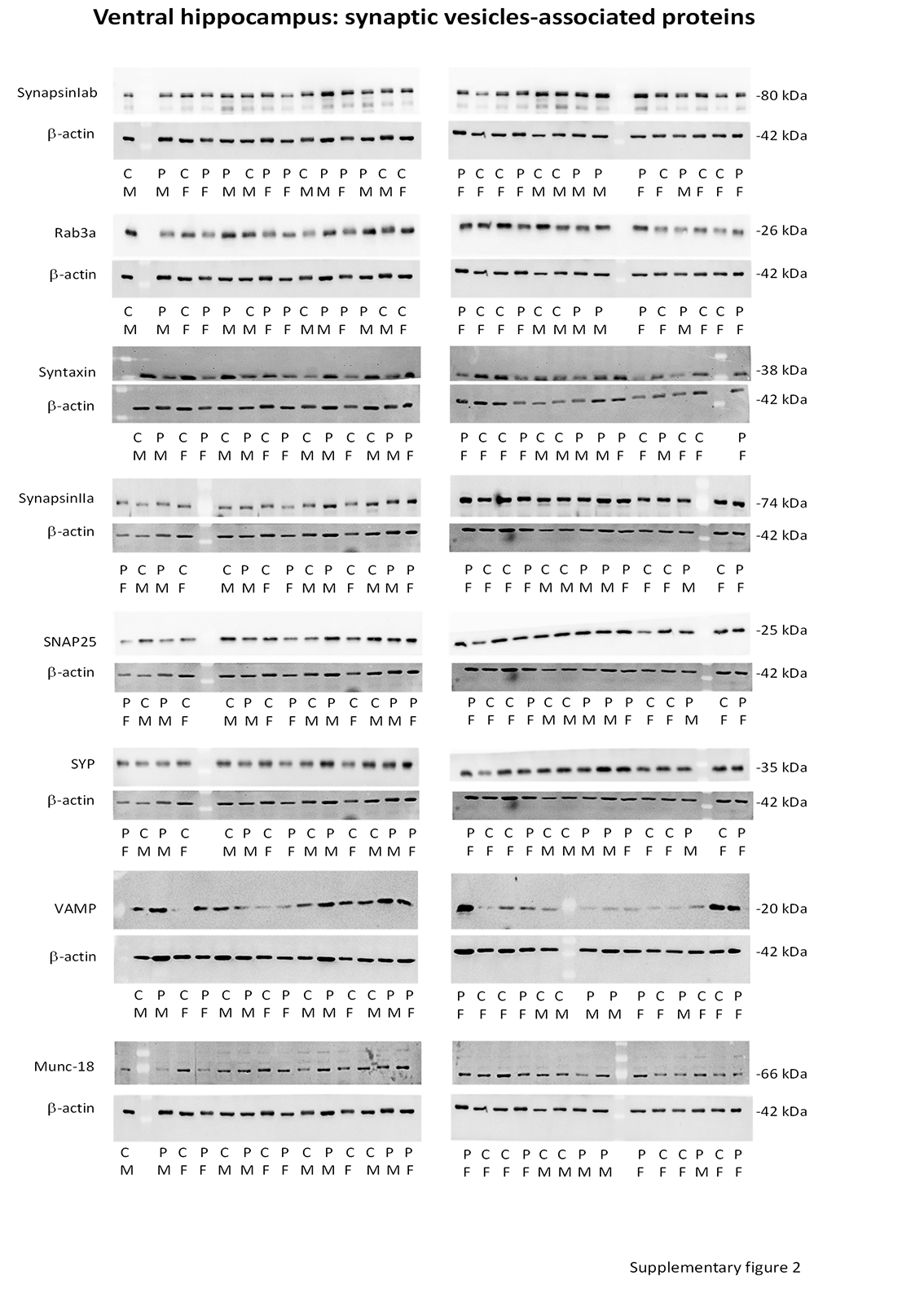

Supplement: Supplementary file 3 — Uncropped images of the immunoblots of the synaptic vesicle-associated proteins in the ventral hippocampus (C: control, P: PRS, M: male, F: female). (PNG 644 kb) [file 11357_2021_375_Fig11_ESM.png]

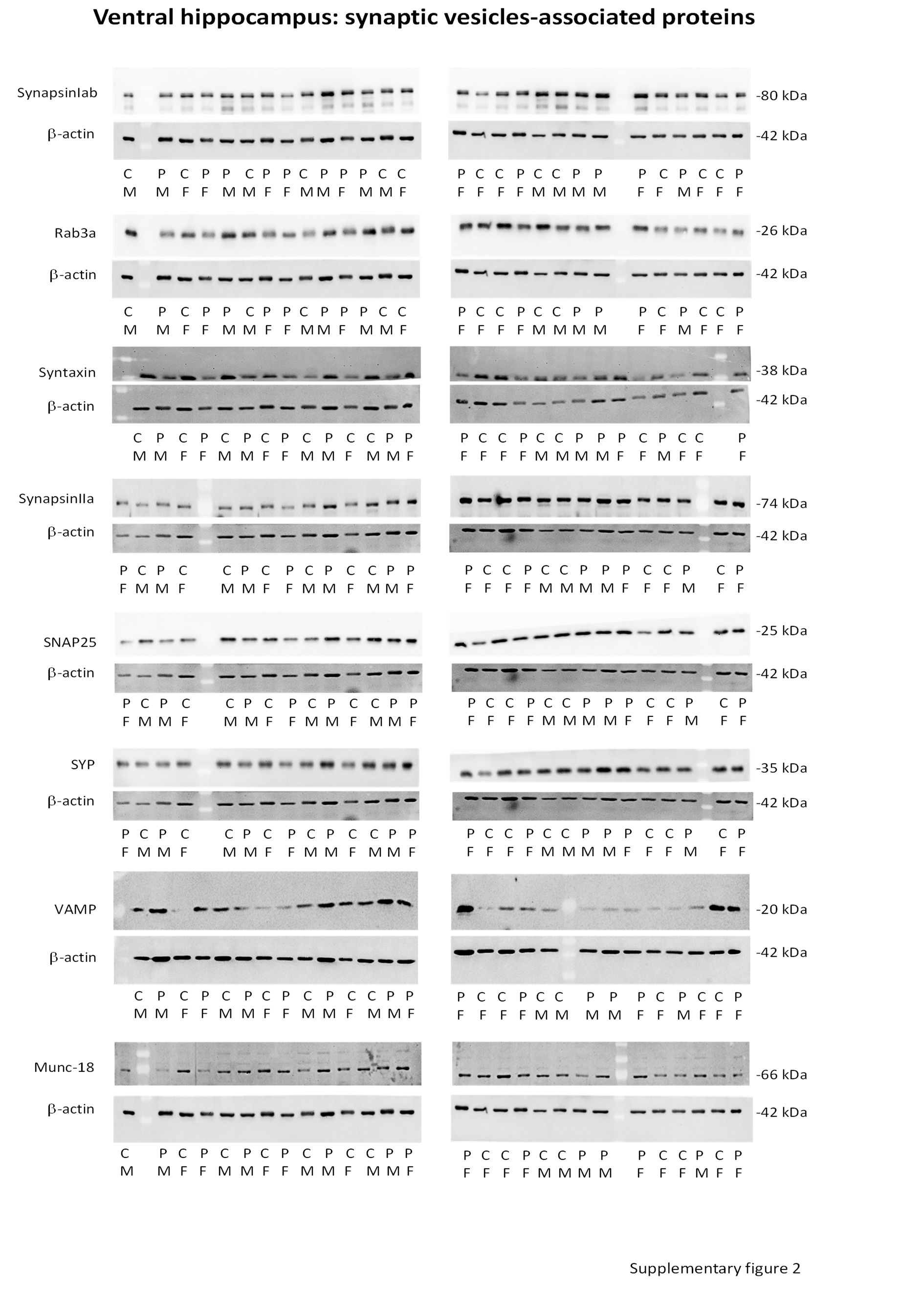

Supplement: Supplementary file 4 — High resolution image (TIF 1740 kb) [file 11357_2021_375_MOESM2_ESM.tif]

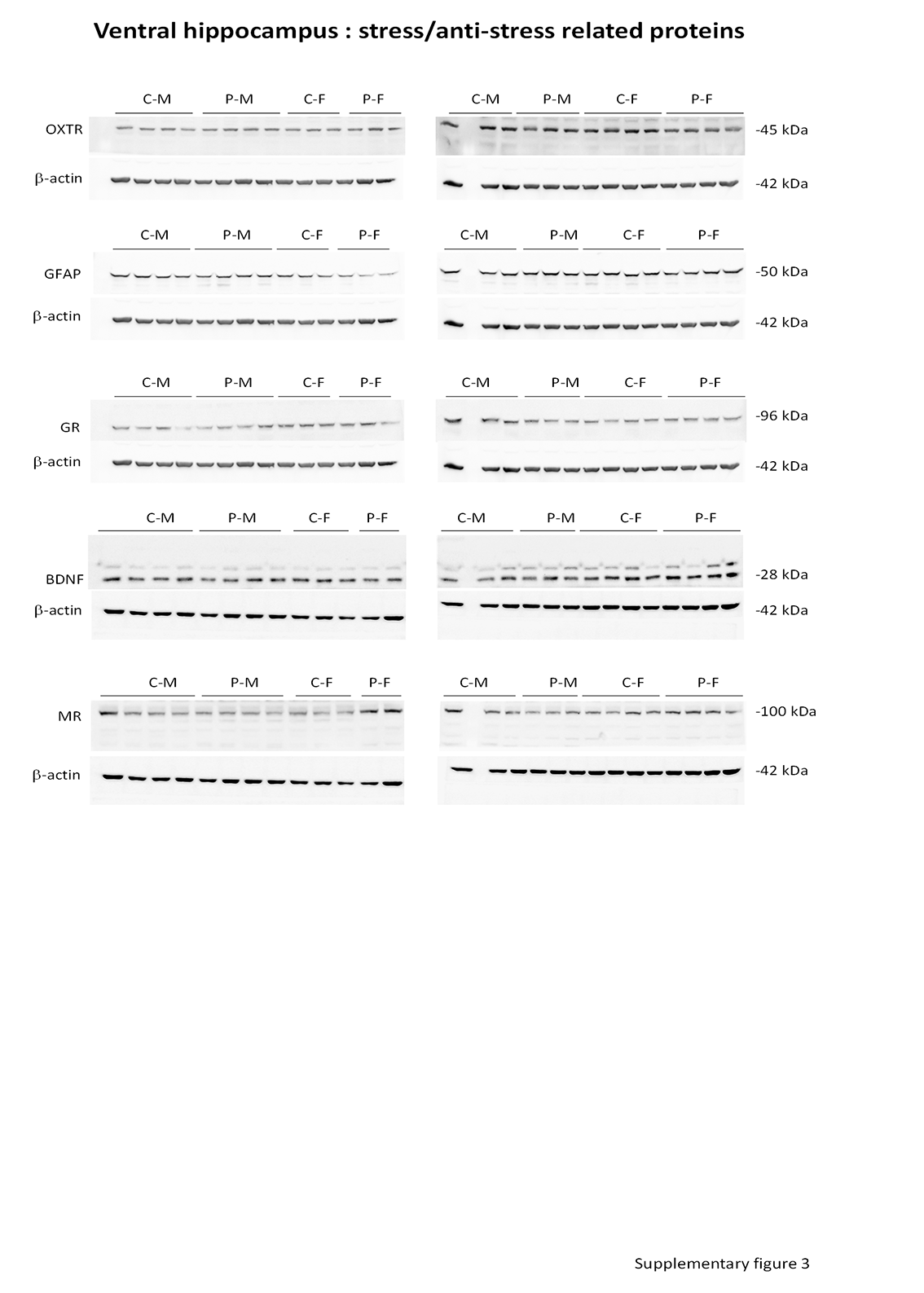

Supplement: Supplementary file 5 — Uncropped images of the immunoblots of the stress/anti-stress related proteins in the ventral hippocampus (C: control, P: PRS, M: male, F: female). (PNG 338 kb) [file 11357_2021_375_Fig12_ESM.png]

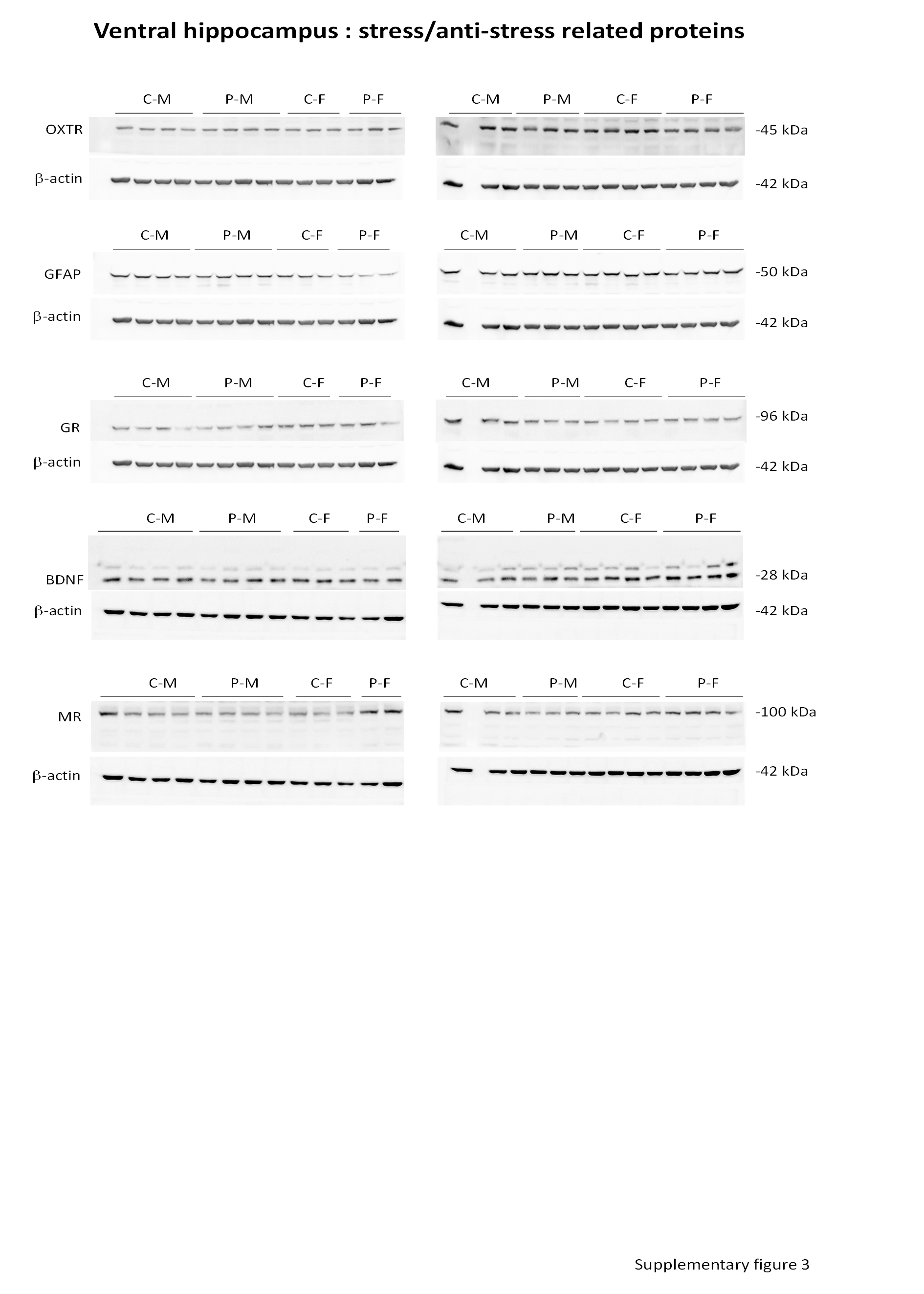

Supplement: Supplementary file 6 — High resolution image (TIF 753 kb) [file 11357_2021_375_MOESM3_ESM.tif]

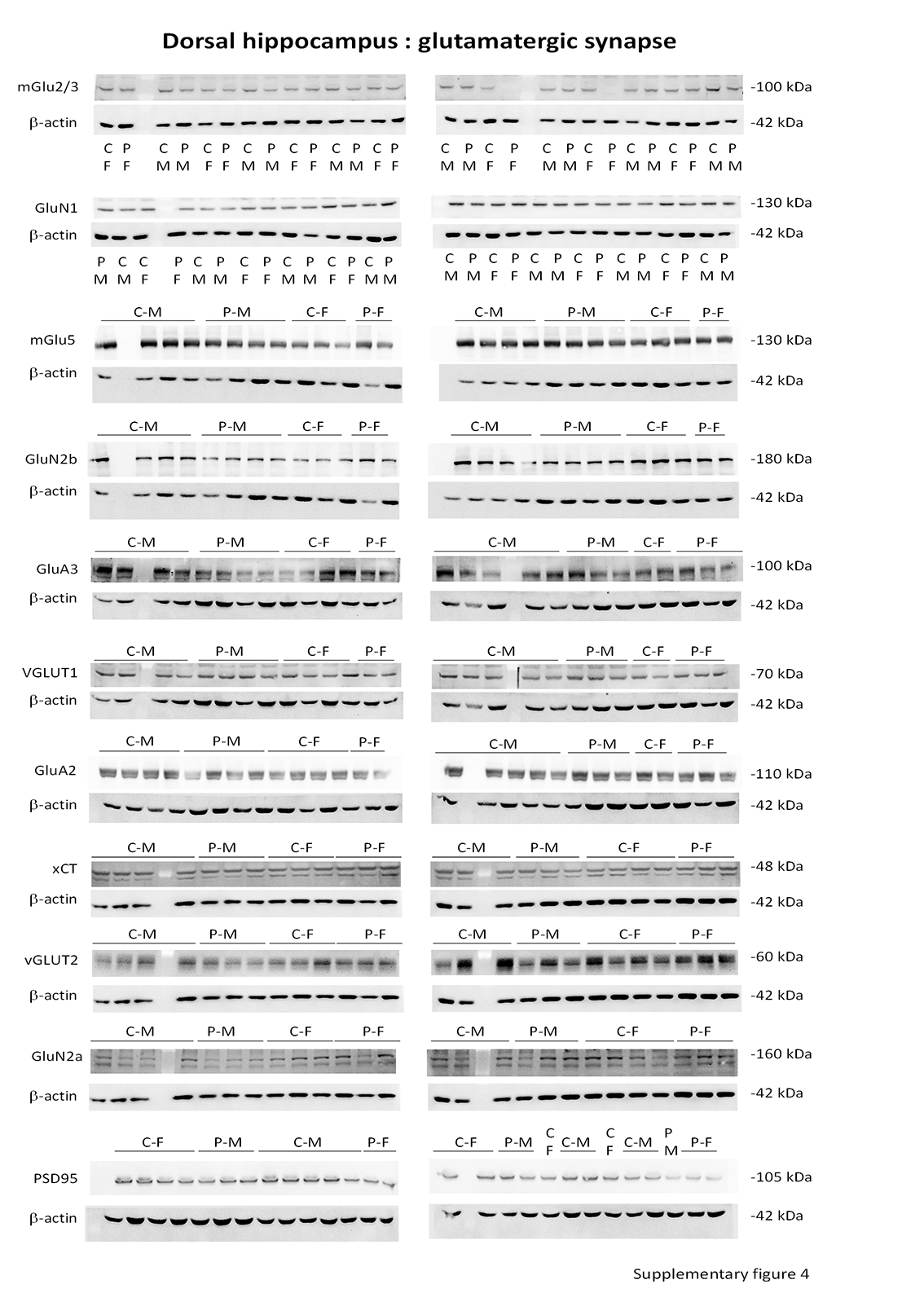

Supplement: Supplementary file 7 — Uncropped images of the immunoblots of the glutamatergic synapse markers in the dorsal hippocampus (C: control, P: PRS, M: male, F: female). (PNG 678 kb) [file 11357_2021_375_Fig13_ESM.png]

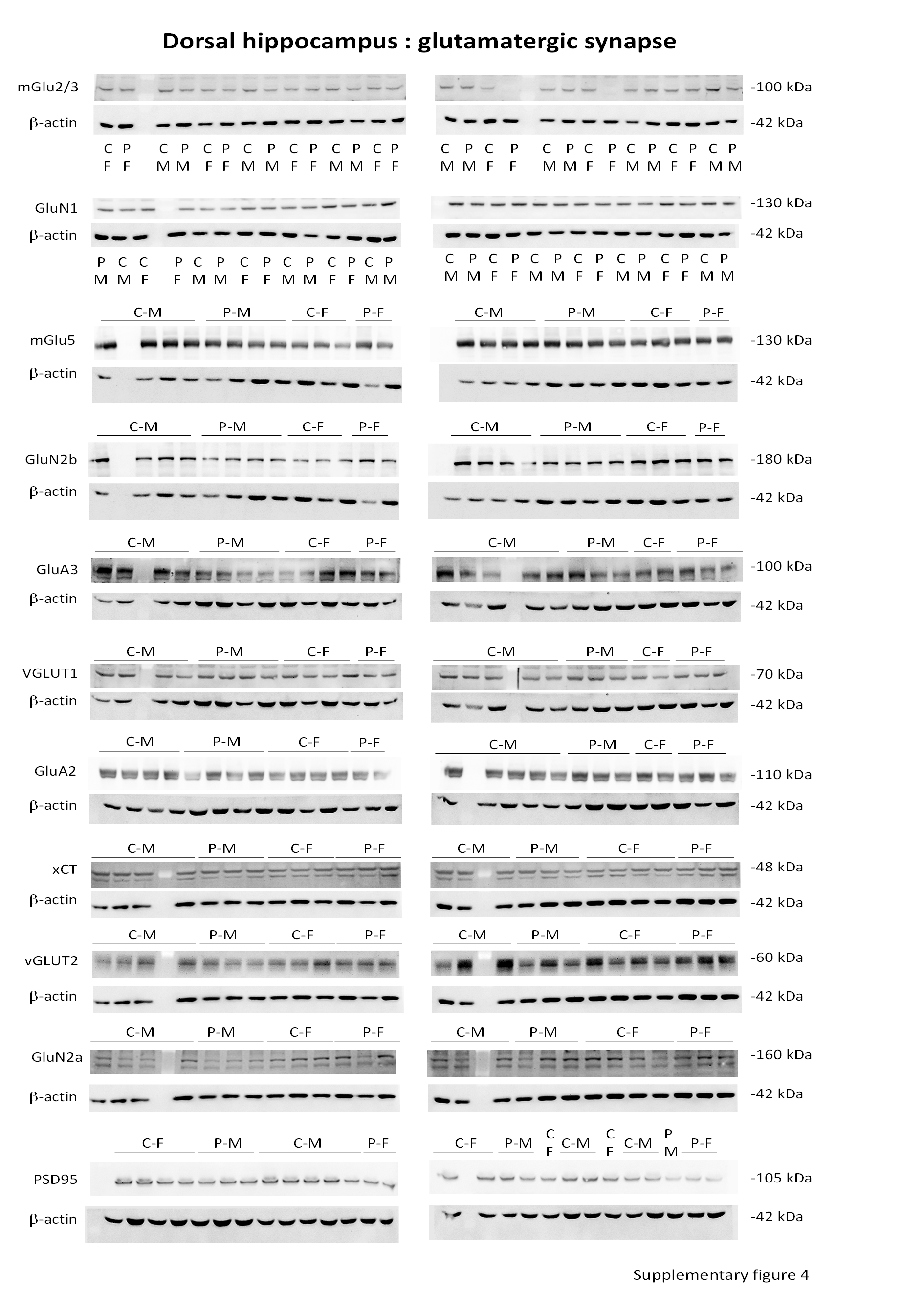

Supplement: Supplementary file 8 — High resolution image (TIF 1631 kb) [file 11357_2021_375_MOESM4_ESM.tif]

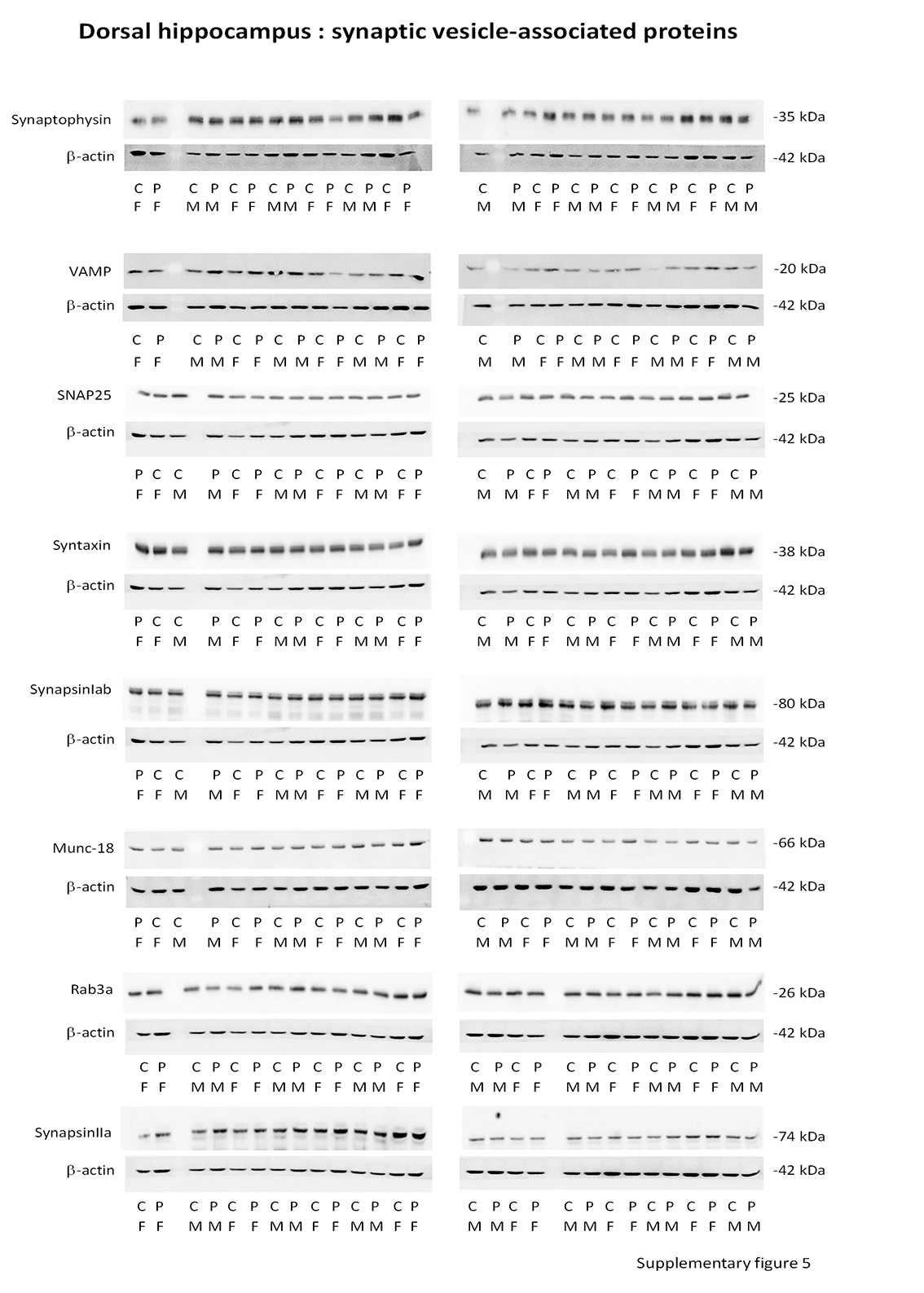

Supplement: Supplementary file 9 — Uncropped images of the immunoblots of the synaptic vesicle-associated proteins in the dorsal hippocampus (C: control, P: PRS, M: male, F: female). (PNG 503 kb) [file 11357_2021_375_Fig14_ESM.png]

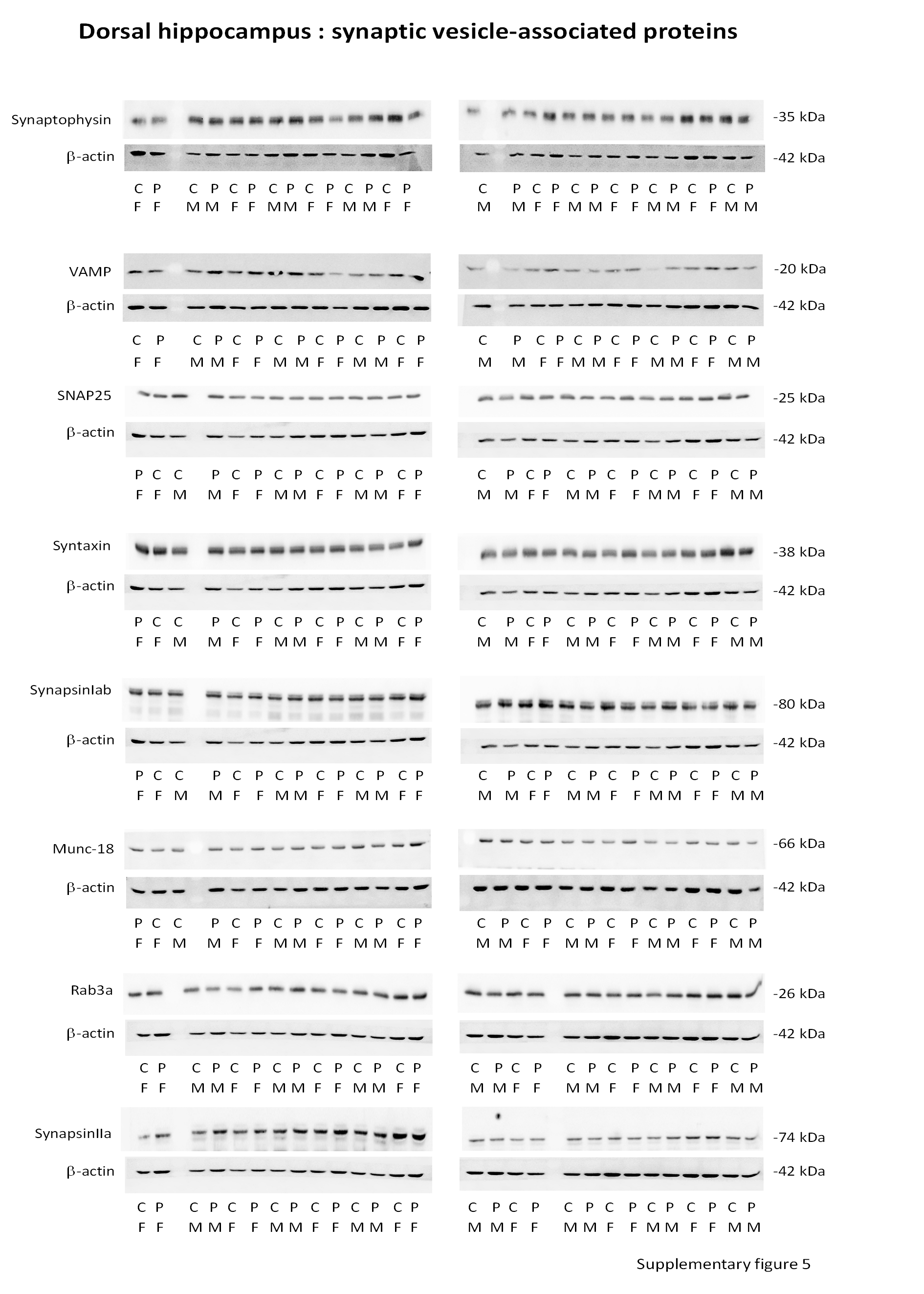

Supplement: Supplementary file 10 — High resolution image (TIF 1195 kb) [file 11357_2021_375_MOESM5_ESM.tif]

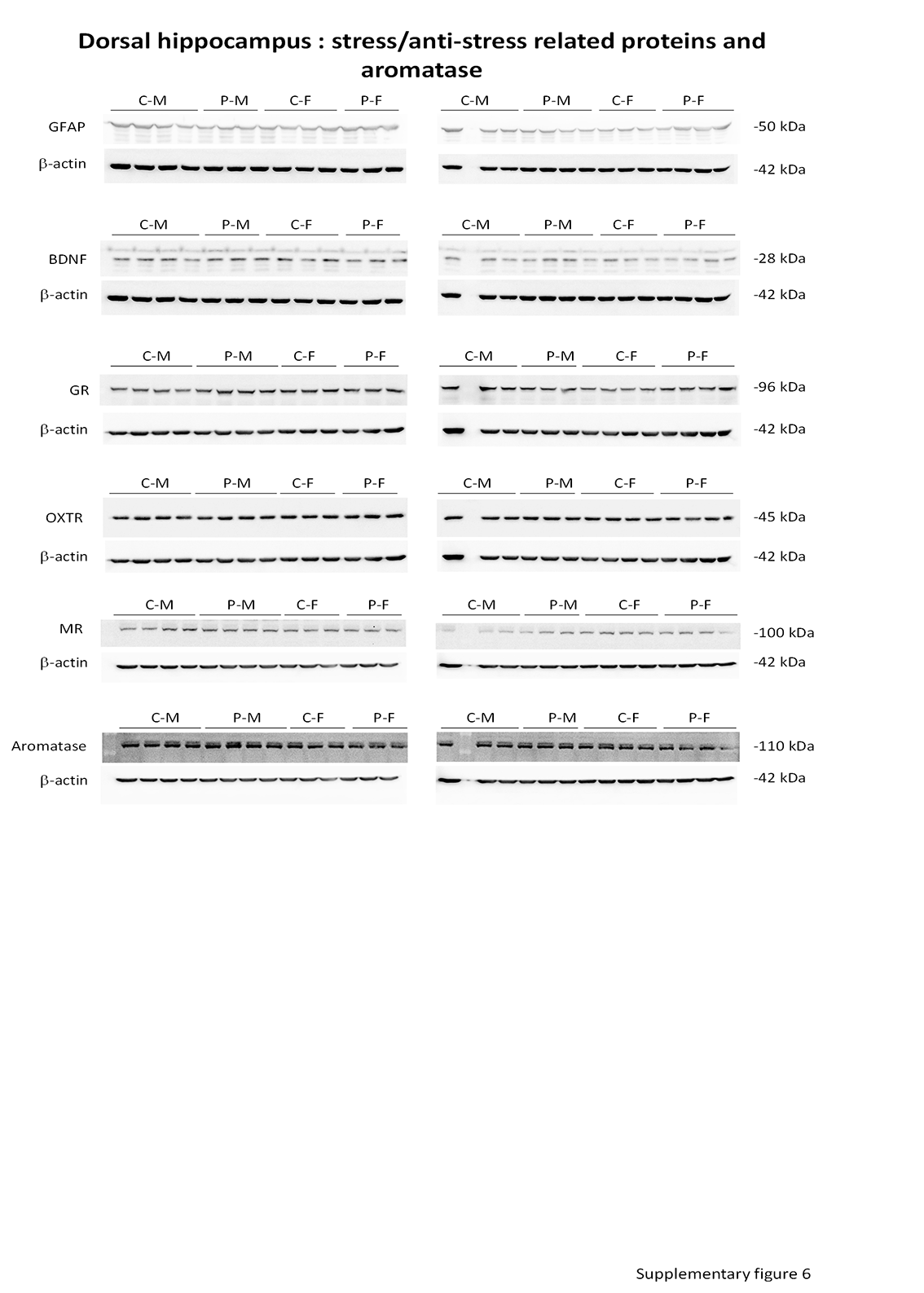

Supplement: Supplementary file 11 — Uncropped images of the immunoblots of the stress/anti-stress related proteins and aromatase in the dorsal hippocampus (C: control, P: PRS, M: male, F: female). (PNG 348 kb) [file 11357_2021_375_Fig15_ESM.png]

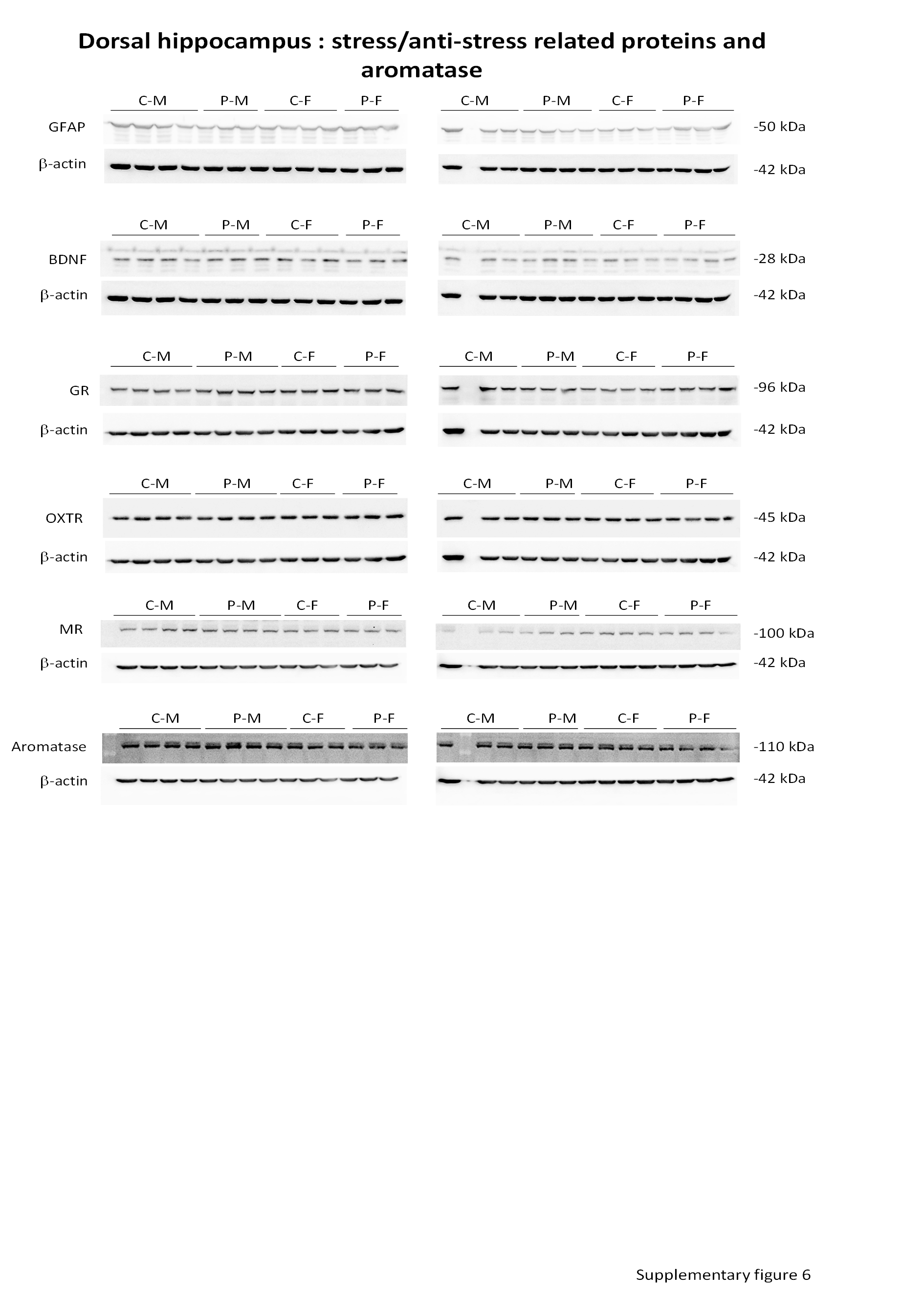

Supplement: Supplementary file 12 — High resolution image (TIF 764 kb) [file 11357_2021_375_MOESM6_ESM.tif]

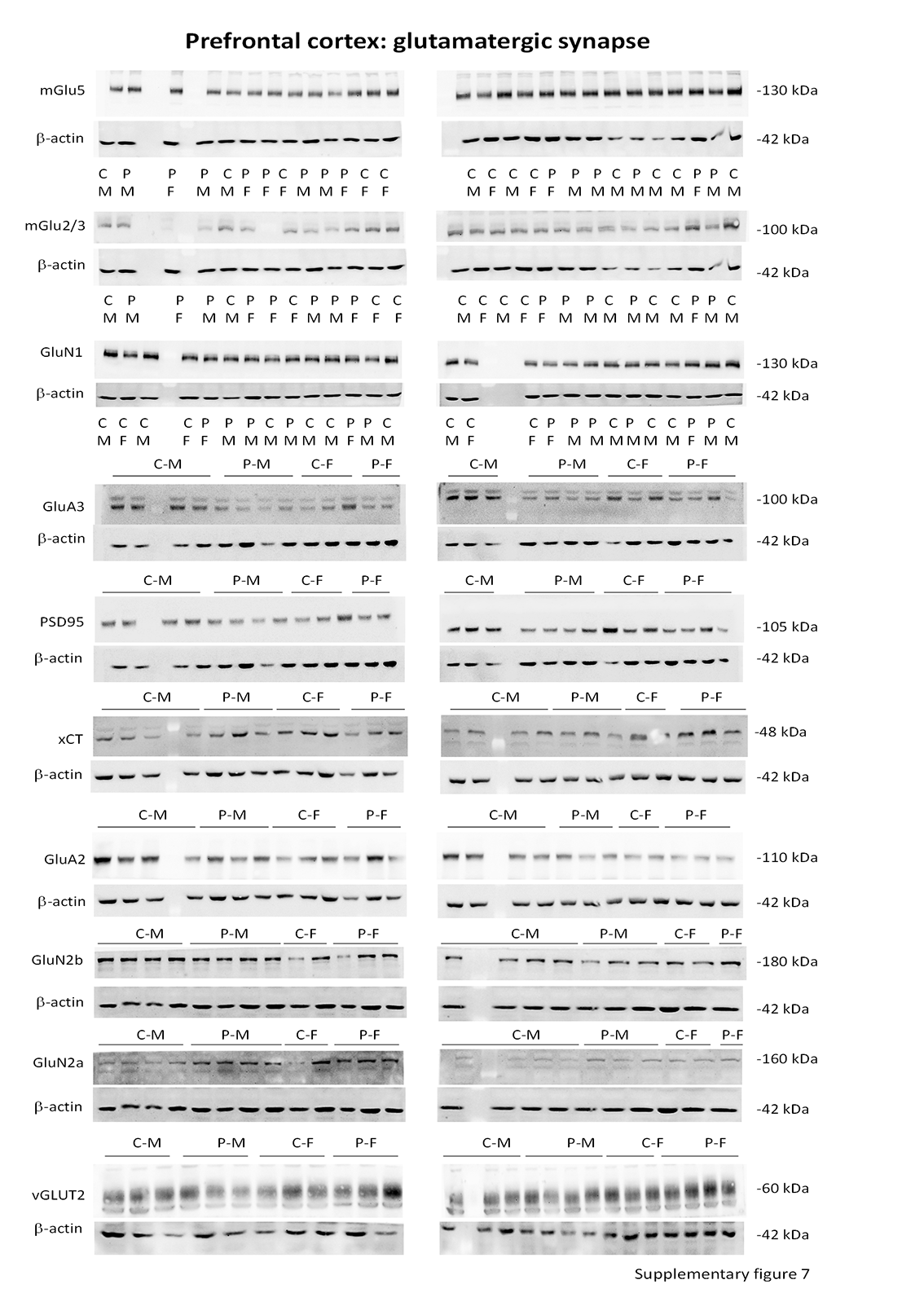

Supplement: Supplementary file 13 — Uncropped images of the immunoblots of the glutamatergic synapse markers in the prefrontal cortex (C: control, P: PRS, M: male, F: female). (PNG 687 kb) [file 11357_2021_375_Fig16_ESM.png]

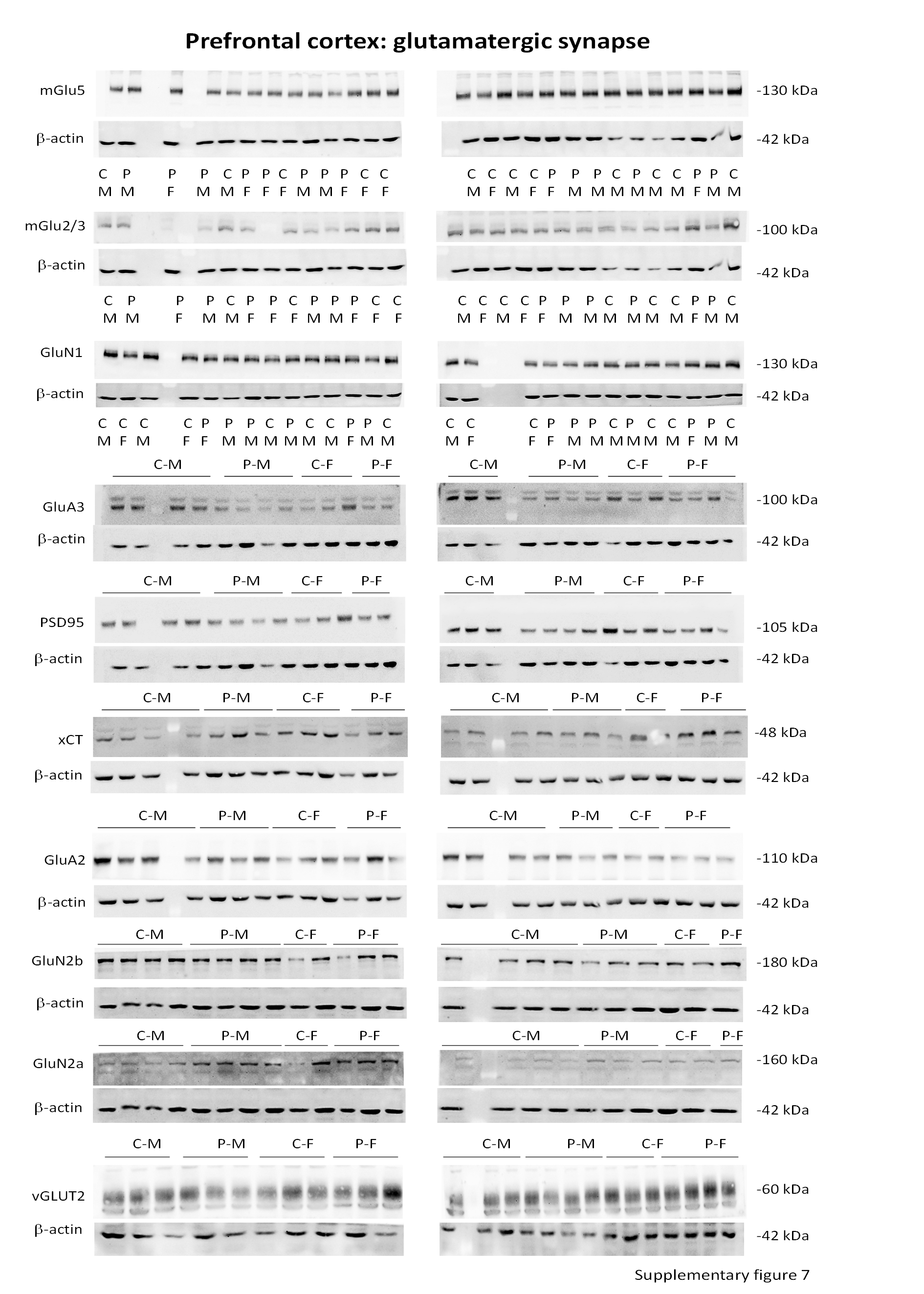

Supplement: Supplementary file 14 — High resolution image (TIF 1714 kb) [file 11357_2021_375_MOESM7_ESM.tif]

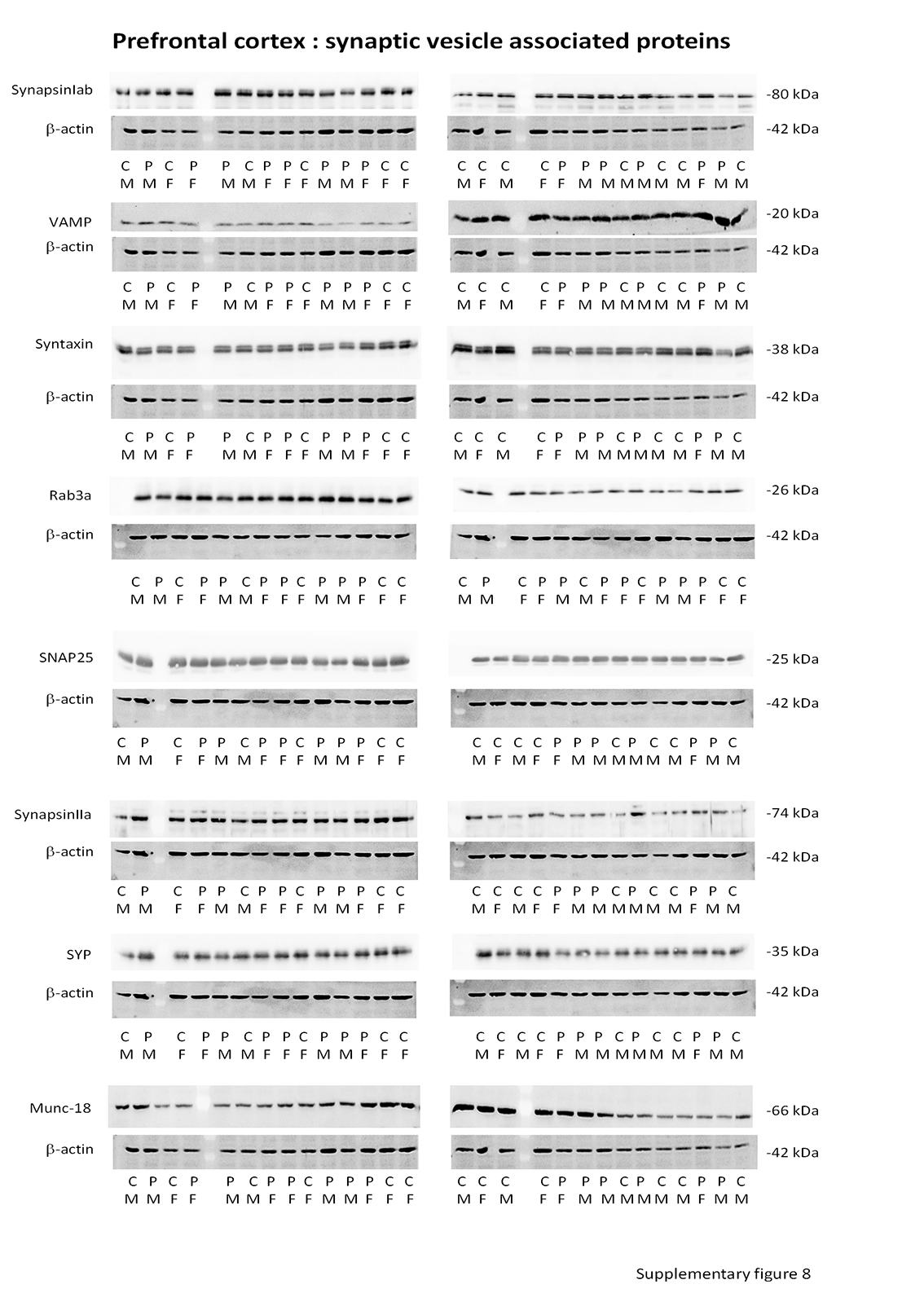

Supplement: Supplementary file 15 — Uncropped images of the immunoblots of the synaptic vesicle-associated proteins in the prefrontal cortex (C: control, P: PRS, M: male, F: female). (PNG 600 kb) [file 11357_2021_375_Fig17_ESM.png]

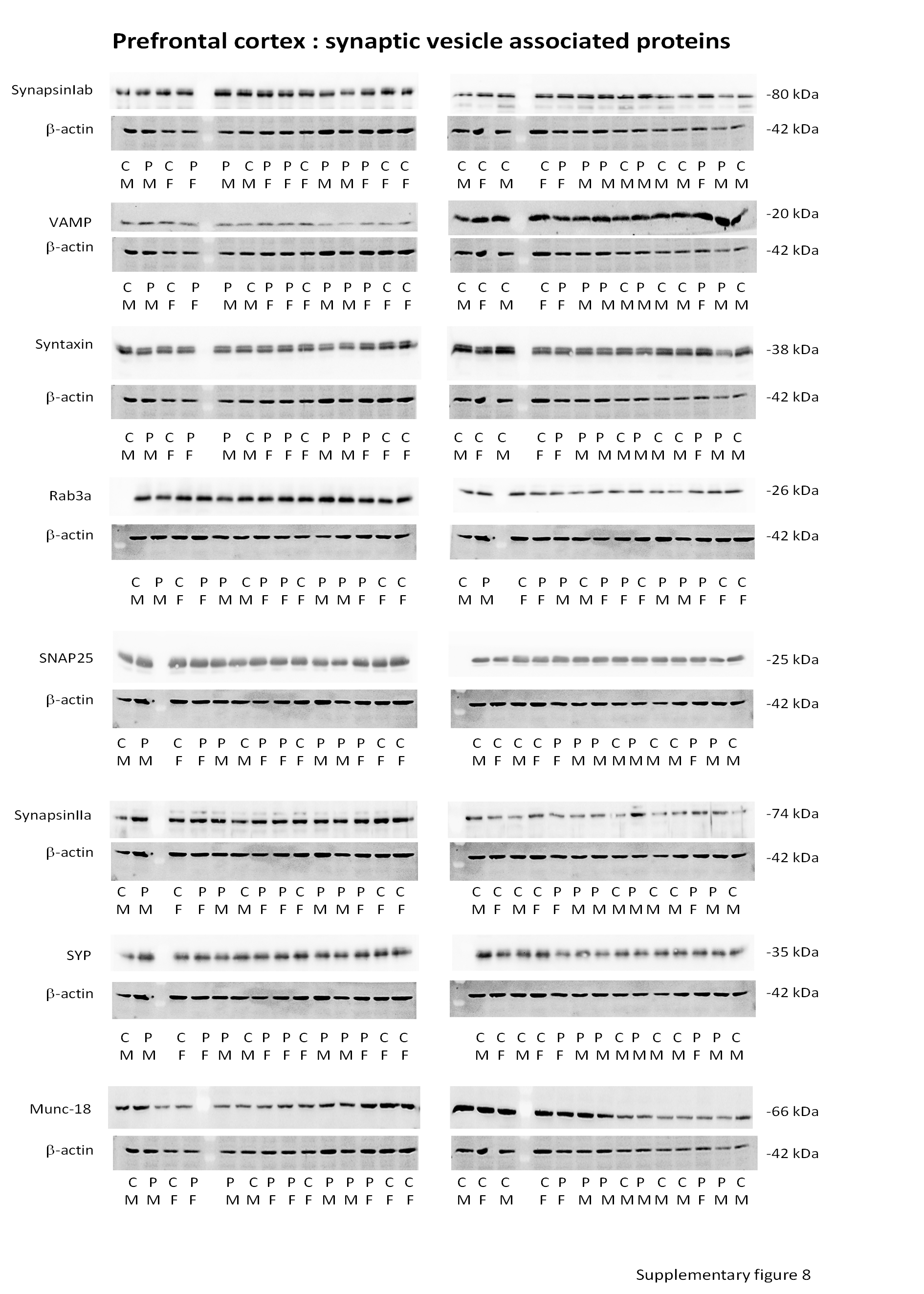

Supplement: Supplementary file 16 — High resolution image (TIF 1501 kb) [file 11357_2021_375_MOESM8_ESM.tif]

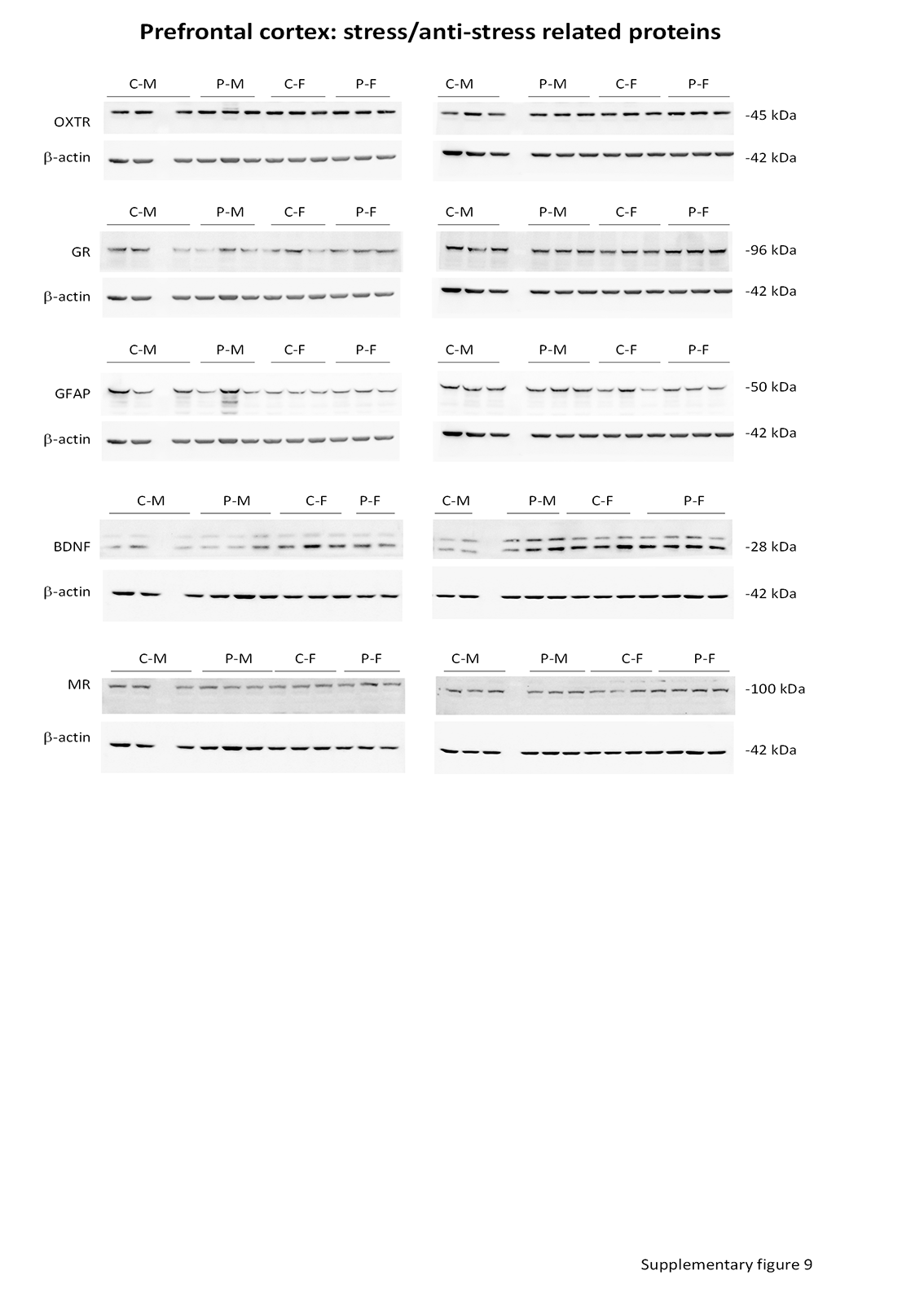

Supplement: Supplementary file 17 — Uncropped images of the immunoblots of the stress/anti-stress related proteins and aromatase in the prefrontal cortex (C: control, P: PRS, M: male, F: female). (PNG 297 kb) [file 11357_2021_375_Fig18_ESM.png]

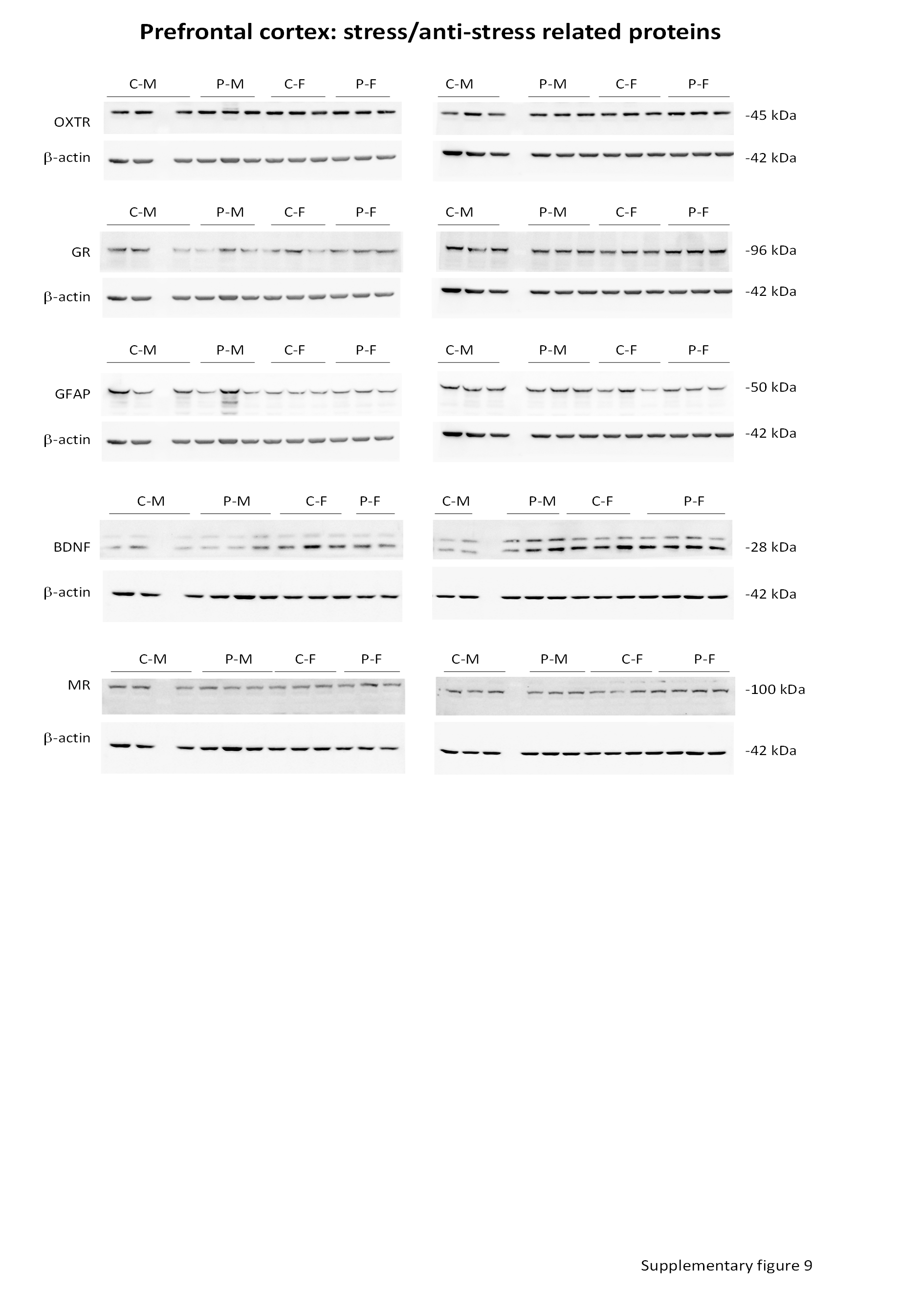

Supplement: Supplementary file 18 — High resolution image (TIF 660 kb) [file 11357_2021_375_MOESM9_ESM.tif]

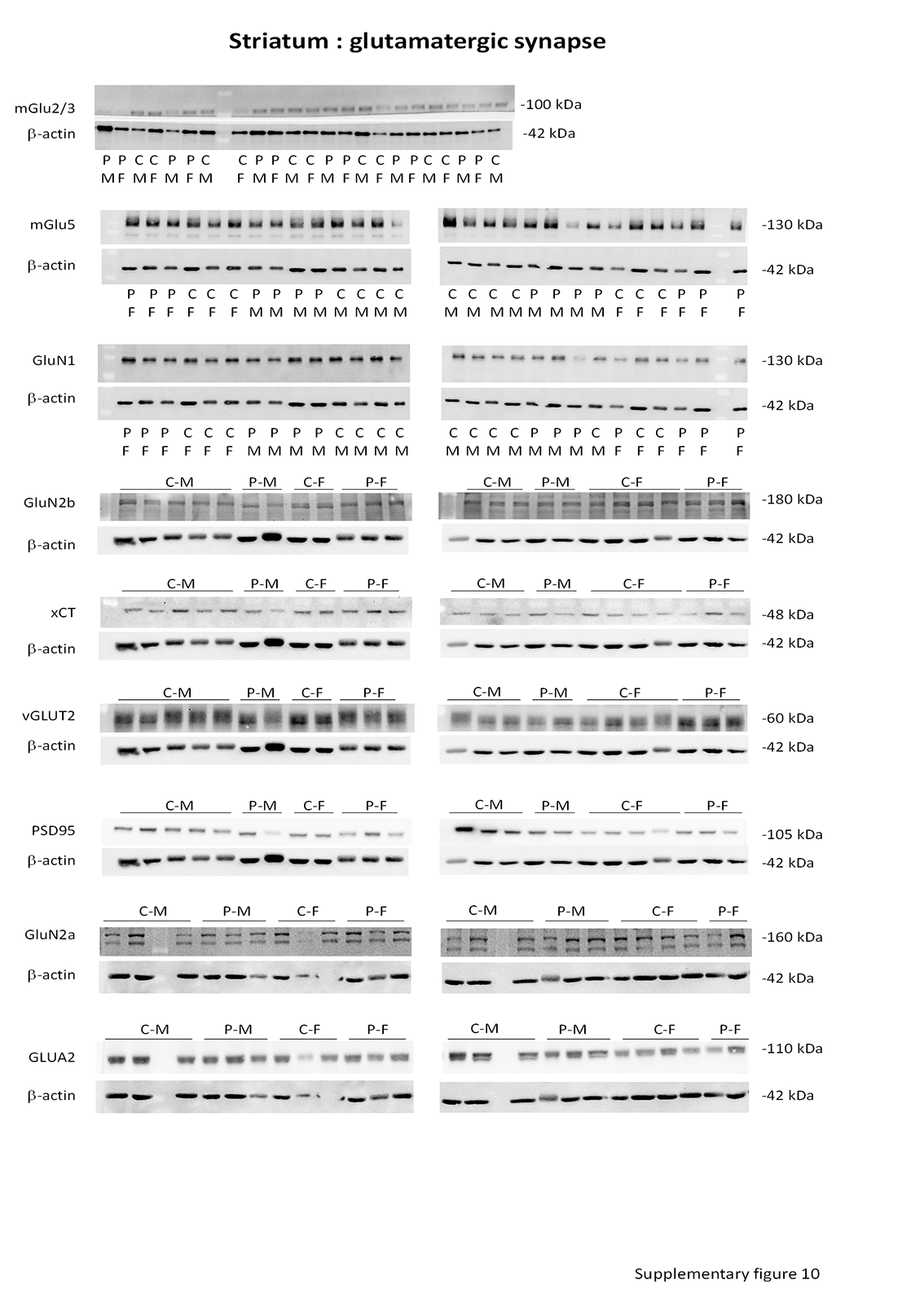

Supplement: Supplementary file 19 — Uncropped images of the immunoblots of the glutamatergic synapse markers in the striatum (C: control, P: PRS, M: male, F: female). (PNG 564 kb) [file 11357_2021_375_Fig19_ESM.png]

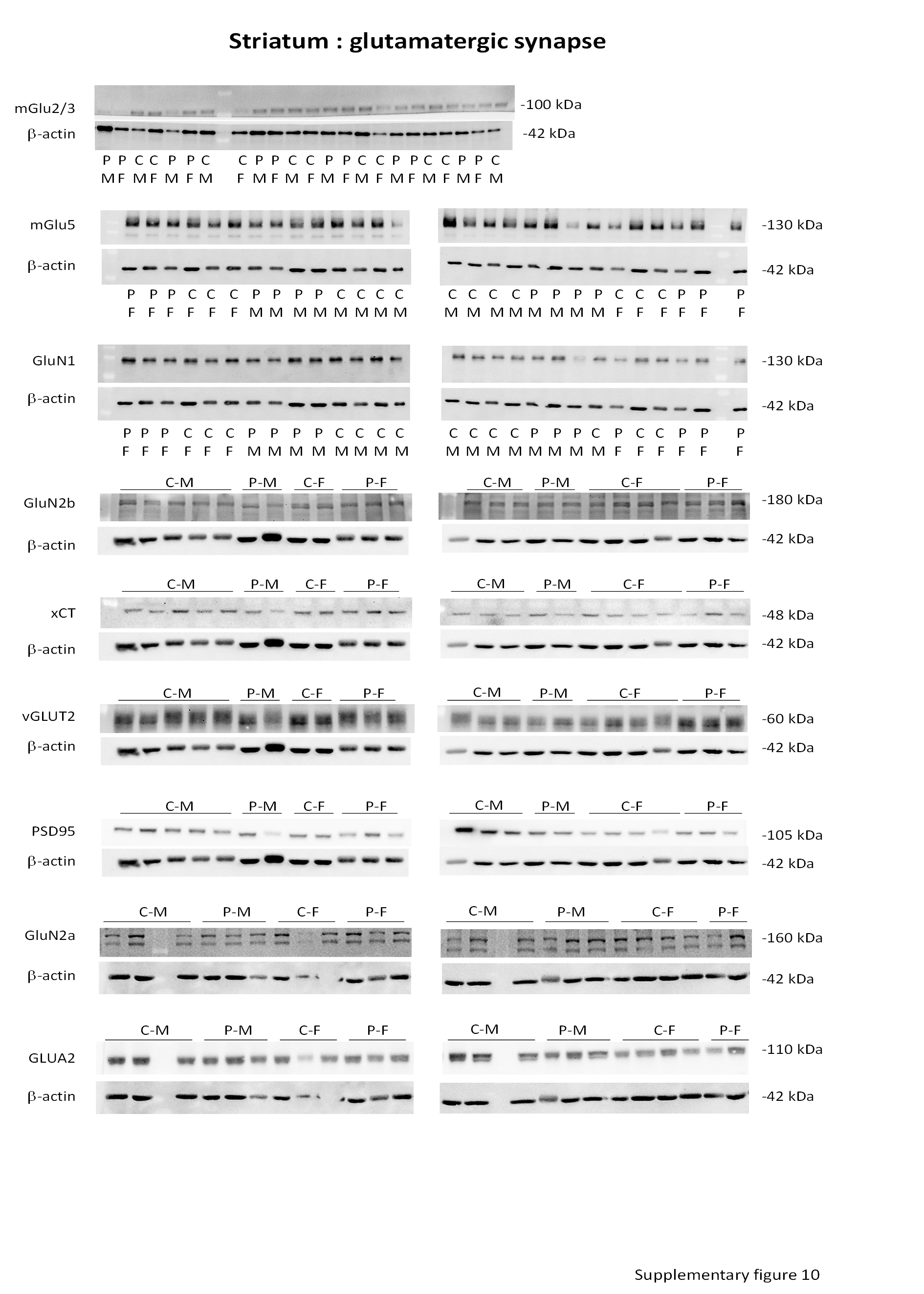

Supplement: Supplementary file 20 — High resolution image (TIF 1400 kb) [file 11357_2021_375_MOESM10_ESM.tif]

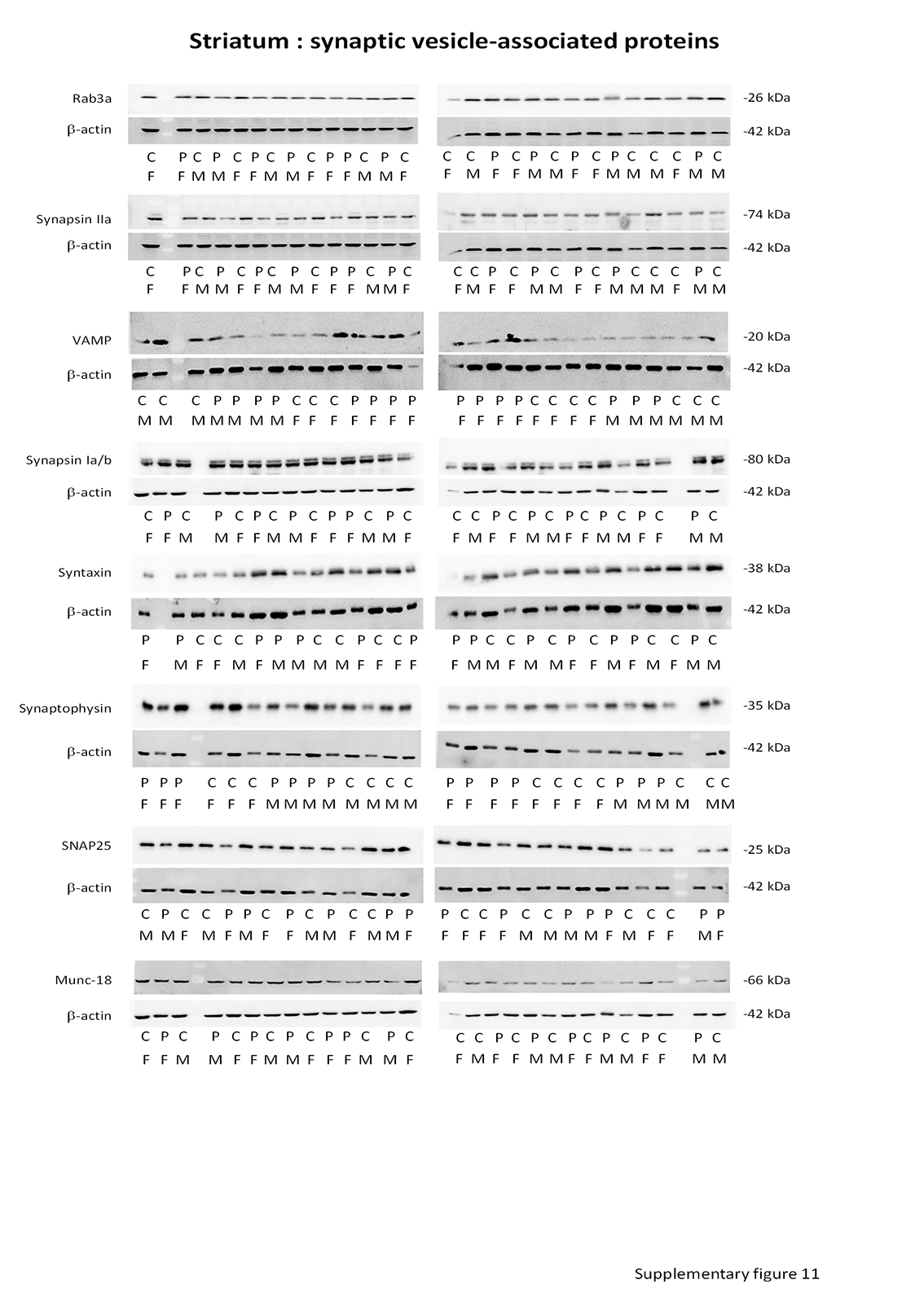

Supplement: Supplementary file 21 — Uncropped images of the immunoblots of the synaptic vesicle-associated proteins in the striatum (C: control, P: PRS, M: male, F: female). (PNG 561 kb) [file 11357_2021_375_Fig20_ESM.png]

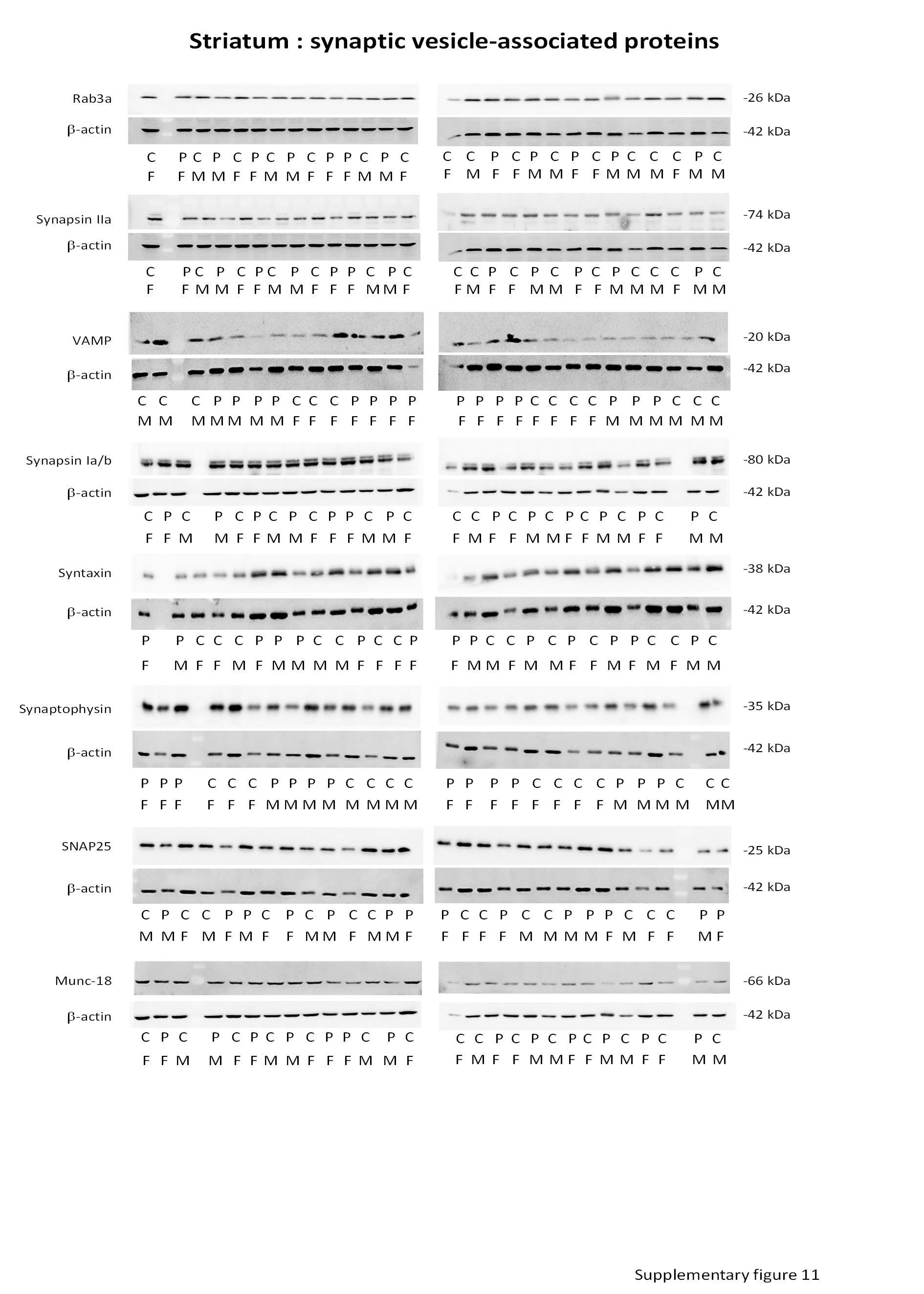

Supplement: Supplementary file 22 — High resolution image (TIF 1358 kb) [file 11357_2021_375_MOESM11_ESM.tif]

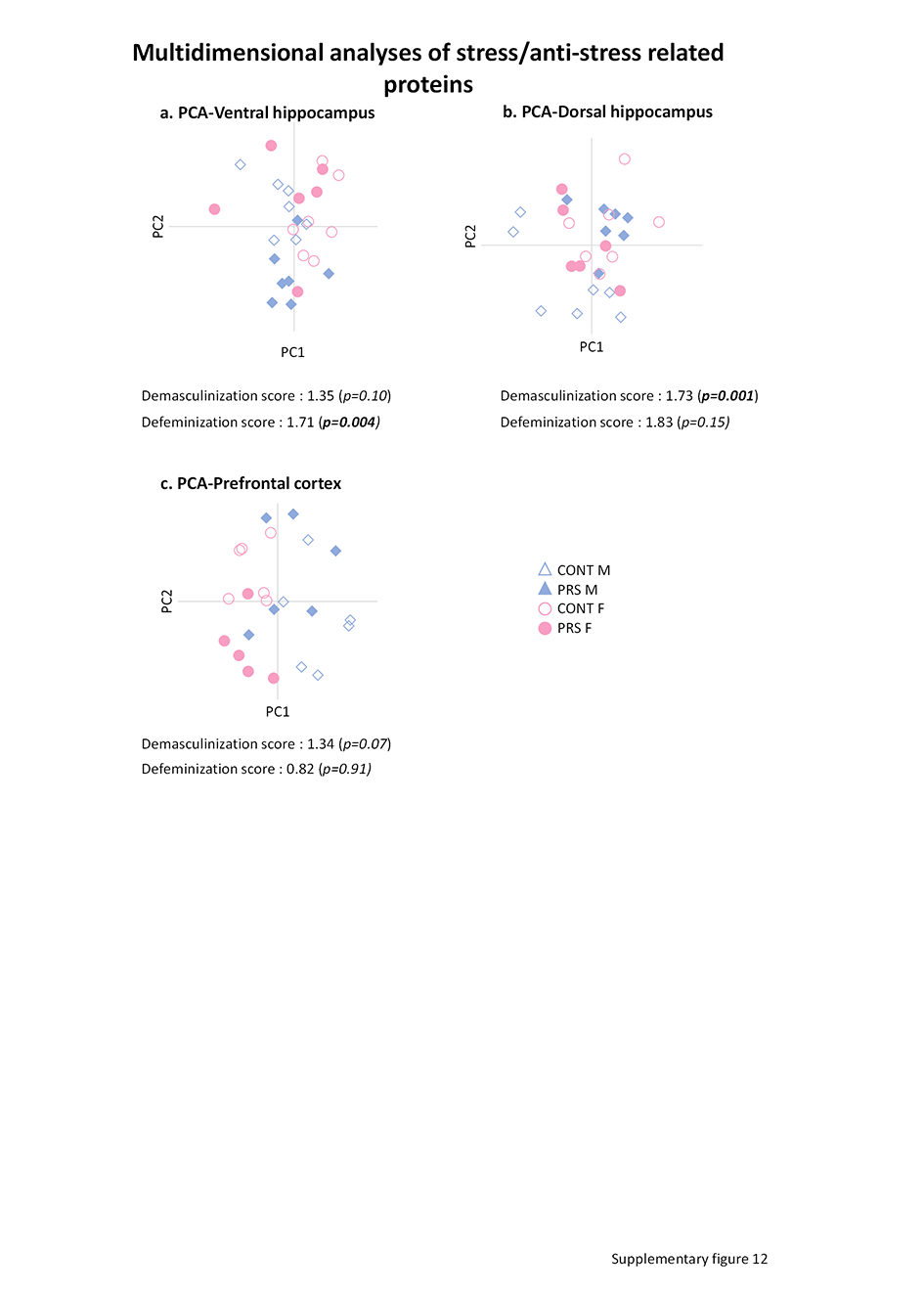

Supplement: Supplementary file 23 — Multidimensional analyses of the stress/anti-stress related proteins in the ventral hippocampus (a), dorsal hippocampus (b), and prefrontal cortex (c). PCA: principal component analysis. (PNG 100 kb) [file 11357_2021_375_Fig21_ESM.png]

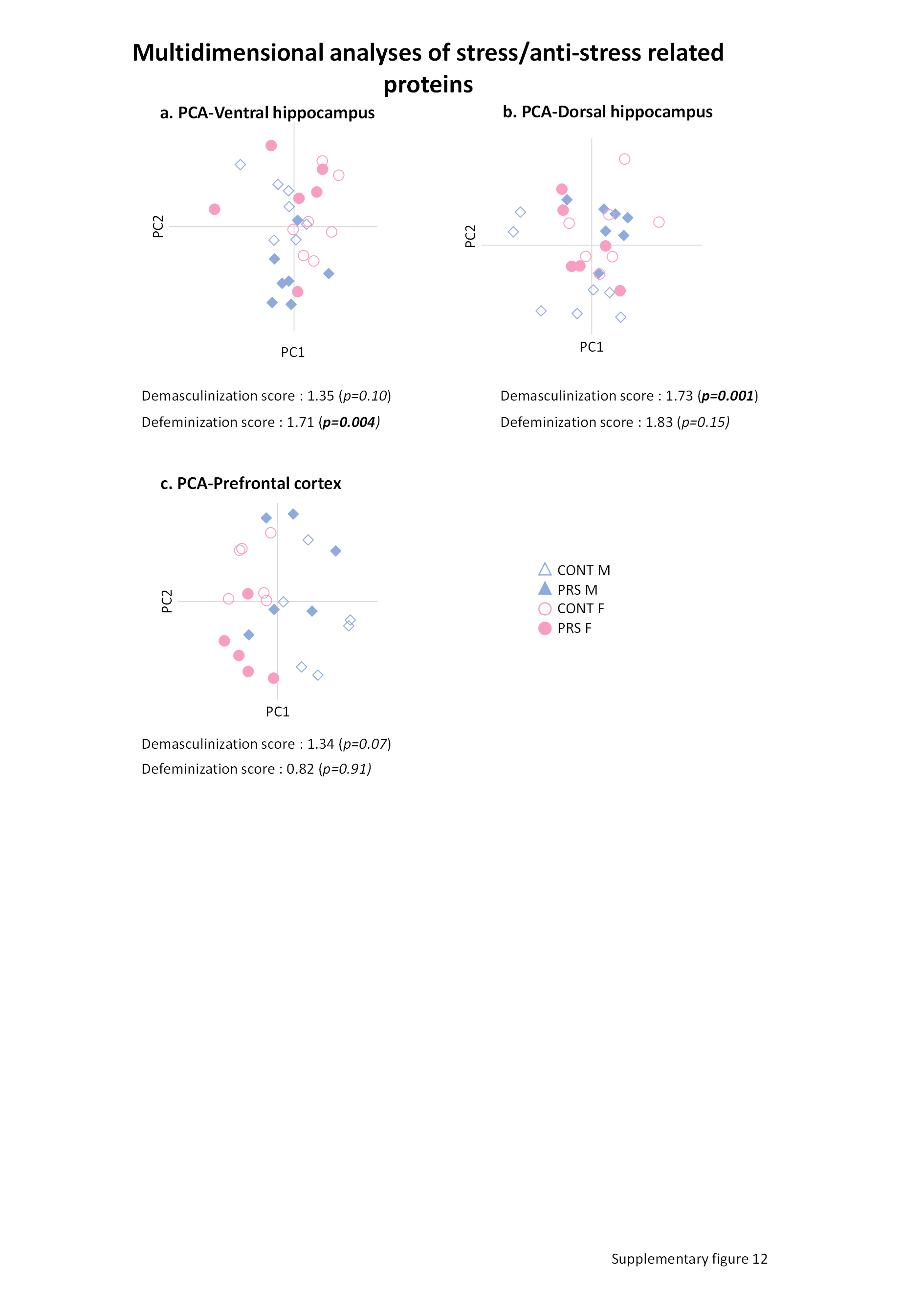

Supplement: Supplementary file 24 — High resolution image (TIF 227 kb) [file 11357_2021_375_MOESM12_ESM.tif]
